# Study of Conversion of Bio-oil Model Compounds in Supercritical Water Using Density Functional Theory

**DOI:** 10.1038/s41598-020-66237-w

**Published:** 2020-06-08

**Authors:** Kushagra Agrawal, Nanda Kishore

**Affiliations:** Department of Chemical Engineering, Indian Institute of Technology Guwahati, Guwahati, Assam 781039 India

**Keywords:** Chemistry, Energy science and technology, Engineering

## Abstract

It is well known that supercritical water is a favourable medium for biomass conversion followed by its hydrodeoxygenation (HDO). Moreover, the actual kinetics and mechanism of reaction occurring in the supercritical water are not yet completely understood, either by experimental or computational approaches. Within the framework of DFT, the major challenge is non-availability of models to simulate supercritical phase. In this study, the authors manually define the descriptors of a solvation model to describe an implicit supercritical phase. In order to examine the suitability of supercritical water for thermal and hydrotreatment of bio-oil model compounds, nine different reactions involving conversion of furfural, tetrahydrofuran, xylose, phenol, guaiacol, ferulic acid, acetic acid, 2-hydroxybenzaldehyde and hydroxyacetone have been considered. Further these reactions are also studied in gas and liquid phase to compare results of different phases, including supercritical water. It was found that while HDO of aromatic compounds like phenol and 2-hydroxybenzaldehyde was favourable in the supercritical phase, smaller molecules like acetic acid and hydroxyacetone did not show much advantage in the supercritical phase over gas and liquid phase. It was also found that the thermochemical parameter - Gibbs free energy change (ΔG) was equally influenced by the solvation effect and the effect of temperature-pressure under supercritical conditions. In several instances, the two effects were found to offset each other in the supercritical phase.

## Introduction

Due to the ever-increasing energy demand and pollution concerns, biomass energy has gained significant importance among researchers as an alternate energy source. This is primarily because the biomass can provide carbon-based fuel which can act as a substitute for petroleum feedstock. But the process of conversion of raw biomass into high-value biofuel and other green chemicals is conventionally very energy-intensive as it is a two-step process^1^. In the first step, the biomass is treated thermally to obtain the depolymerised hydrocarbons in the form of bio-oil. In the next step, this oil is upgraded to match the quality standard of conventional fuel. Hydrodeoxygenation (HDO) is one method of upgrading bio-oil where it is treated with hydrogen to reduce O/C ratio^2^. One approach towards making the process more economical is to treat the biomass with supercritical water. The supercritical water not only provides the ideal temperature and pressure conditions for the conversion but also provides a medium to enhance the kinetic parameters of the upgrading through HDO. Supercritical phase shows several advantages during the production and upgrading of biofuel as it provides a uniform phase where gas and liquid co-exist. This causes better mixing of different species leading to a better heat transfer and mass transfer along with enhanced reaction rate^3,4^. Supercritical solvents are also an eco-friendly solvent as they are non-reactive at normal temperature and pressure conditions. Like hydrothermal liquefaction^5^, supercritical water also offers the advantage of using wet biomass without any pre-treatment.

In modern times, molecular simulation tools are used to determine the thermochemistry and kinetics of reactions. However, the parameters of reactions occurring in supercritical phase are not known till date because the properties of supercritical phase are highly sensitive to pressure and temperature variations. A slight change in temperature or pressure above the critical point leads to a massive change in all properties of the supercritical phase, such as density, dielectric constant, refractive index, aromaticity, etc.

This study sheds some light on the thermochemistry of a few of the reactions occurring during the biofuel formation and upgrading in the supercritical medium. The authors also hope to establish a standard method, for similar but elaborate future computational studies on supercritical fluids, by this work.

## Literature Review

The use of supercritical water for converting organic waste into value-added products was first patented by Modell *et al*.^6^ in 1978. The patent claimed that the organic wastes could be converted into high-value products on treatment with supercritical water, giving a high conversion rate. It was noted that the organic waste underwent cracking, reforming and hydrogenation with or without catalyst. In addition, the formation of char was also noticed to be highly suppressed. This led several researchers to study the use of supercritical water as a solvent for biomass model compound conversion and subsequent upgrading by HDO. For this, Antal *et al*. conducted several experiments^7,8^ to further the understanding of effect of supercritical water as a solvent. In one of their reported studies^7^, biomass was treated at 600 °C and 34.5 MPa in supercritical water with a carbon-based catalyst, and a 100% gasification of the biomass was achieved without any char formation. In another study^8^, glucose was gasified in supercritical phase without any char formation whatsoever. Recently, Matsumara *et al*.^9^ studied the effect of the addition of organic acids to supercritical water towards char suppression, reporting drastic suppression at only ~0.03% addition. Several other works have also studied the effect of supercritical water on bio-fuel synthesis. Savage *et al*.^10^ studied the gasification of phenol and glycine in supercritical water with and without Ni catalyst. They reported a high yield of CO and H_2_ gas in the product. Similarly Goodwin *et al*.^11^ studied the gasification of xylose in water between 450 °C to 650 °C at 250 bar and reported water gas as major product along with methane and ethane. They also proposed kinetic models to describe the gasification. Uemura *et al*.^12^ treated oil palm waste at sub and supercritical temperature. Depending upon the feed, the high bio-oil yield was obtained at 390 °C and 450 °C at 25 MPa and 30 MPa respectively. They attribute the variation in the optimum condition to the different lignocellulose content in the feed. Algal biomass also exhibits better yield on treatment with supercritical water^13^. Shi *et al*.^13^ have shown that by combining hydrothermal liquefaction with supercritical water gasification, the energy recovery from the algal bio-mass can increase by up to 18%.

Computationally, supercritical water reactions have been modelled by several reasearchers to study the gasification of organic matter using molecular dynamics (MD). In a study conducted by Jin *et al*.^14^, the decomposition of anthracene and furfural was studied using Reactive empirical forcefield (ReaxFF) method to determine the product distribution for a reaction of 500 ps. They also tested the effect of Cu and Ni on the production of different entities during the reaction and found that Ni reduced the production of free carbon. In another study^15^ by the same author, gasification of anthracene was studied in a non catalytic system with the same computational tool. It was reported that the supercritical water acted as medium as well as catalyst and reduced the ring breaking energy by 3.5 times. It also led to the formation of small molecules such as CO, CH_4_ and others. The gasification of furfural was also studied by Jing *et al*.^16^ using ReaxFF and DFT method where they determined the product distribution upto 500 ps. They reported that the heterocyclic ring cleaved from the -O-C(CHO)- site and required 266.49 kJ/mol of energy. They also reported that the water clusters enchanced the production of H_2_ and CO in the 700 molecule system at 25 atm pressure. Smilarly, the ReaxFF was used by Li *et al*.^17^ and Han *et al*.^18^ to study the gasification of lignin in supercritical water. Li *et al*.^17^ used the Nimz 3d model of lignin and produced CO_2_, CH_4_ and H_2_ as major products in NVT ensemble. They reported that increase in the density of supercritical water led to the increase in the yield of the products due to increased ionization of the system. Han *et al*.^18^ used Ni as catalyst to study the depolymerisation, fragmentation and hydrogen generation of lignin via the cleavage of the β-O-4′ linkages. They proposed three pathways and studied the product distribution along all the pathway upto 1400 ps. It was reported that the presence of Ni catalyst accelerated the cleavage of C-O bond in the aromatic compounds, and also led to opening of the ring due to destruction of conjugated π bond. The cleavage of C_α_-C_β_ and β-O-4 was also reported by Wang *et al*.^19^ in there MD study for lignin gasification. Gasification of coal in supercritical water has also been modelled by researchers like Zhang^20–22^, Jin^23^, Han^24^, etc. using similar MD methods.

Some efforts have also been made to computationally model biomass degradation and upgrading in supercritical water using quantum mechanics (QM), but they are not very accurate in their approach. For example, in a study conducted by Chen *et al*.^25^, the mechanism of the decomposition of glucose in supercritical water was studied using DFT. But in order to incorporate the supercritical phase effect, the authors^25^ used the PCM solvation model with water solvent and defined the temperature and pressure conditions at supercritical conditions. This is not entirely correct as merely changing the temperature and pressure conditions in the packages do not describe the supercritical phase solvent accurately. In a similar study conducted by Inomata *et al*.^26^, the relative comparison between dehydration reaction and the retro-aldol reaction of glyceraldehyde in supercritical water was reported. The authors used water molecules explicitly and defined the temperature and pressure at supercritical conditions in gas phase. This approach is also not accurate as the contribution of the polarizing supercritical phase which surrounds the entire reactant system is neglected. Thus a comprehensive model, which can define the supercritical phase implicitly, is required to accurately study the influence of supercritical phase on the thermochemistry of the reaction.

In this study, within the framework of DFT, the authors defined the supercritical phase implicitly by defining the solvents using several descriptors and calculated the thermochemistry of conversion of nine reactions which are commonly observed during biomass pyrolysis and upgrading. The nine reactions were chosen such that they covered the variation in -molecular weight,-types of bond, -aromatic and linear groups and -functional groups.

### Supercritical phase descriptors and validation

The ‘universal solvation model based on solute electron density’ or ‘SMD’, developed by Truhlar *et al*.^27^, is one of the most accurate and comprehensive models available to study the solvation effects within the framework of DFT. The SMD model contains an electrostatic component (IEFPCM^28^ with new parameterized atomic radii) and a non-electrostatic component (cavity dispersion solvent structure). The components can be expressed in terms of free energy as:

1 $$\Delta {G}_{S}^{o}=\Delta {G}_{ENP}+\Delta {G}_{CDS}+\Delta {G}_{conc}^{o}$$

where $\Delta {G}_{S}^{o}$ is standard state free energy of solvation, $\Delta {G}_{ENP}$ denotes the electrostatic component of the energy of solvation which includes electronic, nuclear and polarization contributions. $\Delta {G}_{CDS}$ is the cavity-dispersion-solvent structure contribution to the solvation free energy. $\Delta {G}_{conc}^{o}$ is the change in the free energy due to change of concentration between gas phase standard state and liquid phase standard state. The electrostatic effect is incorporated by solving the non-homogenous Poisson’s equation (NPE) which relates the solute charge density $({\rho }_{f})$ to the dielectric constant (ε) with total potential ζ as

2 $${\rm{\nabla }}\cdot (\varepsilon {\rm{\nabla }}\zeta )=-\,4\pi {\rho }_{f}$$

the NPE when solved for the solute (in terms of quantum description) provides electric potential called reaction field (ϕ). The free energy change can then be calculated using the reaction field as

3 $$\Delta {G}_{EP}=\langle \varPsi |{H}^{(0)}-\,\frac{e}{2}{\rm{\phi }}|\varPsi \rangle +\frac{e}{2}{\sum }_{k}{Z}_{k}{{\rm{\phi }}}_{k}-\langle {\varPsi }^{(0)}|{H}^{(0)}|{\varPsi }^{(0)}\rangle $$

where *e* is the unit atomic charge, ${Z}_{k}$ and ${{\rm{\phi }}}_{k}$ are atomic number and reaction field of atom *k* and *H*^*(o)*^ and $\varPsi$^(0)^ are Hamiltonian and electronic wavefunction of solute in gas phase and $\varPsi$ is the polarized electronic wavefunction of solute in the solution. The reaction field inside the molecular cavity for the solute can be defined for any location r as

4 $${\rm{\phi }}({\rm{r}})=\,\sum _{m}\frac{{q}_{m}}{|{\rm{r}}-{{\rm{r}}}_{m}|}$$

where r_*m*_ is the position of any part of surface are on the solute-solvent boundary and *q*_*m*_ is the corresponding charge. The non-electrostatic part, which can also be called cavity-dispersion-solvent structure (CDS) term is the sum of following descriptors:

5 $${G}_{CDS}=\mathop{\sum }\limits_{k}^{atoms}{\sigma }_{k}{A}_{k}({\rm{R}},\{{R}_{{Z}_{k}}+\,{r}_{s}\})+\,{\sigma }^{[{\rm{M}}]}\mathop{\sum }\limits_{k}^{atoms}{A}_{k}({\rm{R}},\{{R}_{{Z}_{k}}+\,{r}_{s}\})\,$$

where R is the geometry parameter for solvent accessible surface area (SASA) which is dependent on set {${R}_{{Z}_{k}}$} of atomic van der Waal’s radii and the solvent radius *r*_*s*_. σ_*k*_, σ^[M]^ and *A*_*k*_ are the atomic surface tension, molecular surface tension and SASA of any atom *k* respectively. The atomic surface tension can be further calculated as:

6 $${\sigma }_{k}=\,{\tilde{\sigma }}_{{Z}_{k}}+\mathop{\sum }\limits_{k{\prime} }^{atoms}\tilde{\sigma }{}_{{Z}_{k}{Z}_{k{\prime} }}{T}_{k}(\{{Z}_{k{\prime} },{R}_{kk{\prime} }\})$$

where ${\tilde{\sigma }}_{Z}$ and ${\tilde{\sigma }}_{ZZ}$ represents the specific parameter for atomic number *k* and *k*′, ${T}_{k}(\{{Z}_{k{\prime} },{R}_{kk{\prime} }\})$ is called cut off tan and is the switching function dependent of the molecular geometry. ${\tilde{\sigma }}_{Z}$ and ${\tilde{\sigma }}_{ZZ}$ can be further calculated using a set of solvent parameters described as

7 $${\tilde{\sigma }}_{i}=\,{\tilde{\sigma }}_{i}^{[n]}n+{\tilde{\sigma }}_{i}^{[\sum {\alpha }_{2}^{H}]}\,\sum {\alpha }_{2}^{H}+{\tilde{\sigma }}_{i}^{[\sum {\alpha }_{2}^{H}]}\sum {\alpha }_{2}^{H}$$

where ${\tilde{\sigma }}_{i}$ is either ${\tilde{\sigma }}_{Z}$ or ${\tilde{\sigma }}_{ZZ}$, n is the refractive index of solvent at room temperature, $\sum {\alpha }_{2}^{H}$ is the Abraham’s hydrogen bond acidity and ${\sum {\beta }_{2}^{H}}^{}$ is the Abraham’s hydrogen bond basicity with corresponding empirical parameters. The molecular surface tension ${\sigma }^{[{\rm{M}}]}$ is also dependent on a set of descriptors and is calculated as:

8 $${\sigma }^{[{\rm{M}}]}={\tilde{\sigma }}^{[\gamma ]}\left(\frac{\gamma }{{\gamma }_{o}}\right)+\,{\tilde{\sigma }}^{[{\phi }^{2}]}{\phi }^{2}+{\tilde{\sigma }}^{[{\psi }^{2}]}{\psi }^{2}+{\tilde{\sigma }}^{[{(\sum {\beta }_{2}^{H})}^{2}]}{(\sum {\beta }_{2}^{H})}^{2}$$

where *γ* is the macroscopic surface tension of the solvent at air-solvent interface at room temperature, *ϕ* is fraction of solvent atoms which are aromatic carbon, $\psi$ is the fraction of solvent atoms which are halogens (F, Cl and Br) and $\sum {\beta }_{2}^{H}$ is the Abraham’s hydrogen bond basicity with corresponding empirical constants.

For this study, SMD solvation model of Gaussian 09 package^29^ along with visualization package GaussView 05^30^ were used. SMD model requires a total of seven descriptors, which have been standardized at near room temperature, to incorporate the electrostatic component and a non-electrostatic component of the solvation system^31^. These seven descriptors are dielectric constant (ε), refractive index (*n*), Abraham’s hydrogen bond acidity $(\sum {\alpha }_{2}^{H})$, Abraham’s hydrogen bond basicity $(\sum {\beta }_{2}^{H})$, macroscopic surface tension (γ), aromaticity (ϕ) and electronegative halogenicity (ψ). Thus, in order to define the supercritical water solvent, each of these descriptors were manually provided by the authors as user defined input conditions. In other words, despite the standardization of SMD model descriptors at near room temperature, the descriptors were defined manually at the supercritical conditions in the solver to obtain reaction energies at supercritical conditions. This is required because the properties of supercritical water tremendously vary in the supercritical region from liquid water even for a minute change in temperature and pressure of supercritical phase. From the literature^32,33^ it was observed that the dielectric constant and refractive index show strong correlation to the density of the water above its critical point. Thus, density was chosen as one primary variable instead of two (temperature and pressure) variables to define all parameters. At the desired temperature and pressure condition in the supercritical region, the density of water was calculated using WolframAlpha water density calculator^34^. The calculator uses the ‘International Association for the Properties of Water and Steam (IAPWS) Formulation 1995’^35^ method which is based on the dimensionless Helmholtz free energy (*f*) of water with the relation of the form:

9 $$\frac{f(\rho ,\,T)}{RT}={\Phi }^{\circ }(\delta ,\tau )+{\Phi }^{r}(\delta ,\tau )$$

where ${\Phi }^{\circ }(\delta ,\tau )$ is the ideal gas contribution and ${\Phi }^{r}(\delta ,\tau )$ is the deviation due to real behaviour of fluid. And $\delta$ = *ρ*/*ρ*_*c*_ and $\tau$ = *T*_*c*_/*T* where *ρ*_*c*_ is the critical density and *T*_*c*_ is the critical temperature of water and *ρ* and *T* are density and temperature of water, R is the universal gas constant. The ideal gas part of the equation is calculated as^36^:

10 $${\Phi }^{\circ }=\,\mathrm{ln}\,\delta +{n}_{1}^{o}+{n}_{2}^{o}\tau +{n}_{3}^{o}ln\tau +\mathop{\sum }\limits_{i=4}^{8}{n}_{i}^{o}ln[1-{e}^{-{\gamma }_{i}^{o}\tau }]$$

where ${n}_{i}^{o}$ and ${\gamma }_{i}^{o}\,$are numerical coefficients^37^. Similarly, The real part of the equation is calculated as:

11 $${\Phi }^{r}=\mathop{\sum }\limits_{i=1}^{7}{n}_{i}{\delta }^{{d}_{i}}{\tau }^{{t}_{i}}+\mathop{\sum }\limits_{i=8}^{51}{n}_{i}{\delta }^{{d}_{i}}{\tau }^{{t}_{i}}{e}^{-{\delta }^{{c}_{i}}}+\mathop{\sum }\limits_{i=52}^{54}{n}_{i}{\delta }^{{d}_{i}}{\tau }^{{t}_{i}}{e}^{-{\alpha }_{i}{(\delta -{{\epsilon }}_{i})}^{2}-{\beta }_{i}{(\tau -{\gamma }_{i})}^{2}}\,+\mathop{\sum }\limits_{i=55}^{56}{n}_{i}{\Delta }^{{b}_{i}}\delta {\rm{\psi }}$$

where $\Delta ={\theta }^{2}+{B}_{i}{[{(\delta -1)}^{2}]}^{{a}_{i}}$, $\theta =(1-\tau )+{A}_{i}{[{(\delta -1)}^{2}]}^{\frac{1}{2{\beta }_{i}}}$ and $\psi =\,{e}^{-{C}_{i}{(\delta -1)}^{2}-{D}_{i}{(\tau -1)}^{2}}$ with ${A}_{i},{B}_{i},{\alpha }_{i},{\beta }_{i},{c}_{i},$ ${d}_{i},{t}_{i},{n}_{i}$ and ${\gamma }_{i}$ as numerical coefficients obtained from fitting experimental data^37^.

The derivative of ${\Phi }^{r}$ with respect to *δ* gives ${\Phi }_{\delta }^{r}$ which is related to the pressure (P) as^35^:

12 $$P=(1+{\Phi }_{\delta }^{r}.\delta )(\rho RT)$$

Equation (12) is then used to back calculate the density of water. The dielectric constant values were then obtained from the works of Uematsu & Franck^32^ and refractive index values were obtained from the works of Harvey *et al*.^33^ at the calculated density.

In early attempts to define solvent parameters, Kamlet and Taft came up with solvatochromatic parameters^38,39^ which reflected the ease of formation of hydrogen bond (α and β) between solute and solvent. Over time, these parameters were refined by Abraham^40^ who called it hydrogen bond acidity and basicity (α_2_ and β_2_). However, the parameters put forth by Abraham, also had some shortcomings and were eventually redefined as summation scales of solute hydrogen-bond acidity $(\sum {{\rm{\alpha }}}_{2}^{{\rm{H}}})$ and basicity $(\sum {{\rm{\beta }}}_{2}^{{\rm{H}}})$. In this regard, an interesting observation was noted by Abboud and co-worker^41^ who reported that there exist close relation between the solvent and gas phase hydrogen bond acidity and basicity (α_2_, β_2_). Based on this finding, Abraham concluded that, if required, α_2_ and β_2_ can also be used in gas phase calculations^42^. By this conclusion, the authors assumed that the hydrogen bond acidity and basicity parameters are an inherent property of solvent compound and remain largely independent of the phase of the compound. Based on this assumption, $\sum {{\rm{\alpha }}}_{2}^{{\rm{H}}}$ and $\sum {{\rm{\beta }}}_{2}^{{\rm{H}}}$ of supercritical water was taken to be constants in this study with $\sum {{\rm{\alpha }}}_{2}^{{\rm{H}}}$ = 0.82 and $\sum {{\rm{\beta }}}_{2}^{{\rm{H}}}$ = 0.35^42^. Since one of the characteristic features of supercritical phase is uniformity in the phase with no distinction between gas and liquid phase boundary, the macroscopic surface tension (γ) parameter was also taken to be 0 for the study. Also, since water does not contain any halogen or aromatic atoms, ψ and ϕ were defined as 0.

In order to validate the parameters, the authors reproduced the work of Melius *et al*.^43^. Melius *et al*. simulated water gas shift reaction above critical condition in the gas phase at BAC-MP4 level of theory. Then they calculated the effect of supercritical phase by accounting for the deviation from ideal gas law using the Peng-Robinson equation of state. A departure function was added to the ideal gas temperature dependent difference in Gibbs free energy term and a pressure-dependent difference in Gibbs free energy term to obtain the supercritical phase free energy change. This reaction mechanism proposed by Melius *et al*. was simulated in this study at M06-2X/6-311 + g(d,p) level of theory. The reaction temperature was defined as 700 K and pressure as 300 atm, which was identical to the reported conditions by Melius *et al*.^43^. SMD implicit solvation model was used, and each descriptor was manually defined, as explained above. The difference between the reported value and this work was no more than ±3.11 kcal/mol (see Table 1), which was well within the DFT error limits. Further, to determine Gibbs free energy of the reaction, popular general functionals which are commonly employed for organics systems were tested. It was also observed that M06-2X and M05-2X performed equally well for the water gas shift reaction in supercritical phase and corresponding comparison with the results of Melius *et al*.^43^ is shown in Table 2. Finally, since the SMD model was parameterized using M05-2X functional by its creators, the authors decided to continue with M05-2X for all further simulations.

**Table 1:** Comparison of the results of water gas shift reaction in the supercritical phase.

	Energy for CO + H_2_O → HCOOH (TS1) (kcal/mol)	Energy for HCOOH → CO_2_ + H_2_ (TS2) (kcal/mol)
Melius *et al*.^43^	61.70	64.90
Present study	64.14	61.74

**Table 2:** Different functional and basis sets comparison for ΔG of TS1 and TS2 for water gas shift reactions and their comparison.

Trial	Functional/basis set	ΔG of TS1 at 700 K, 300 atm (kcal/mol)	ΔG of TS2 at 700 K, 300 atm (kcal/mol)
Gas (298 K, 1 atm)	M06-2X/6-311 + g(d,p)	67.01	66.10
Supercritical phase	M06/6-311 + g(d,p)	57.31	66.40
Supercritical phase	M06-2X/6-311 + g(d,p)	65.05	73.25
Supercritical phase	M05-2X/6-311 + g(d,p)	66.86	71.79
Supercritical phase	M06-2X/6-311 + g(d,p)//M052x/6-311 + g(d,p)	63.25	71.64
Supercritical phase	M06-2X/6-31 g(d)	65.63	69.91
Supercritical phase	B3LYP/6-311 + g(d,p)	56.17	67.71

Once the method for parameter defining was standardized, nine different reactions which are commonly observed during HDO of bio-oil were simulated at six different reaction conditions. M05-2X/6-311 + g(d,p) level of theory was used for geometry optimization, frequency calculation and single point energy calculation for all the reactants and products regardless of the medium. This was done to study the behaviour of the supercritical phase towards different type of reactions during pyrolysis of biomass and its upgrading. Of the six conditions studied, four conditions were taken in the supercritical region, one condition was taken below the boiling point of the heaviest compound to study the reaction in liquid phase, and one condition was taken above the boiling point of the heaviest compounds in the gas phase. The liquid phase simulations were performed with the SMD solvation model with water as the solvent. The coordinates of the optimized molecular structures of the reactants and products in all phases are provided in Supplementary Information in Table 1S. The four supercritical region simulations (SC1, SC2, SC3 and SC4) were performed at ρ = 0.089 g/cc, 0.109 g/cc, 0.190 g/cc and 0.360 g/cc of supercritical water, respectively. Since the properties of the supercritical phase near the critical region are extremely sensitive to any variation in operating parameters, the four points were chosen slightly away from the critical point (Fig. 2). The corresponding solvent descriptor values were defined as given in Table 3.

**Table 3:** Parameters and descriptor values of the four supercritical conditions considered in the study.

Condition	Temperature (K)	Pressure (atm)	Density (g/cc)	^a^Dielectric constant (ε)	^b^Refractive index (*n*)	^c^Abraham’s Hydrogen bond acidity ($\sum {\alpha }_{2}^{H}$)	^d^Abraham’s Hydrogen bond basicity ($\sum {\beta }_{2}^{H}$)	Macroscopic surface tension (γ)	Aromaticity (ϕ)	Halogenicity (ψ)
SC1	773	246.73	0.0894	1.500	1.0634	0.82	0.35	0	0	0
SC2	723	246.73	0.109	1.745	1.0861	0.82	0.35	0	0	0
SC3	700	300.00	0.190	3.955	1.1504	0.82	0.35	0	0	0
SC4	723	457.00	0.360	5.478	1.2467	0.82	0.35	0	0	0

^a^Dielectric constant values taken from the work of Uematsu & Franck^32^.

^b^Refractive index values taken from the work of Harvey *et al*.^33^.

^c^Abraham’s Hydrogen bond acidity values taken from the work of Abraham^42^.

^d^Abraham’s Hydrogen bond basicity values taken from the work of Abraham^42^.

The thermodynamic parameters were finally calculated from the optimized geometry by calculating the partial second derivative of the energy along the nuclear coordinates. From the obtained force constants, vibrational frequencies were calculated using harmonic approximations. These vibrational frequencies were then used to derive the thermal corrections to the energy to provide entropy and enthalpies of the structure^44^. The entropy and enthalpy were then used to calculate the Gibbs free energy as per the equations:

13 $$\Delta {\rm{G}}=\Delta {\rm{H}}\text{-}{\rm{T}}\Delta {\rm{S}}$$

## Reactions

**Figure 1:**
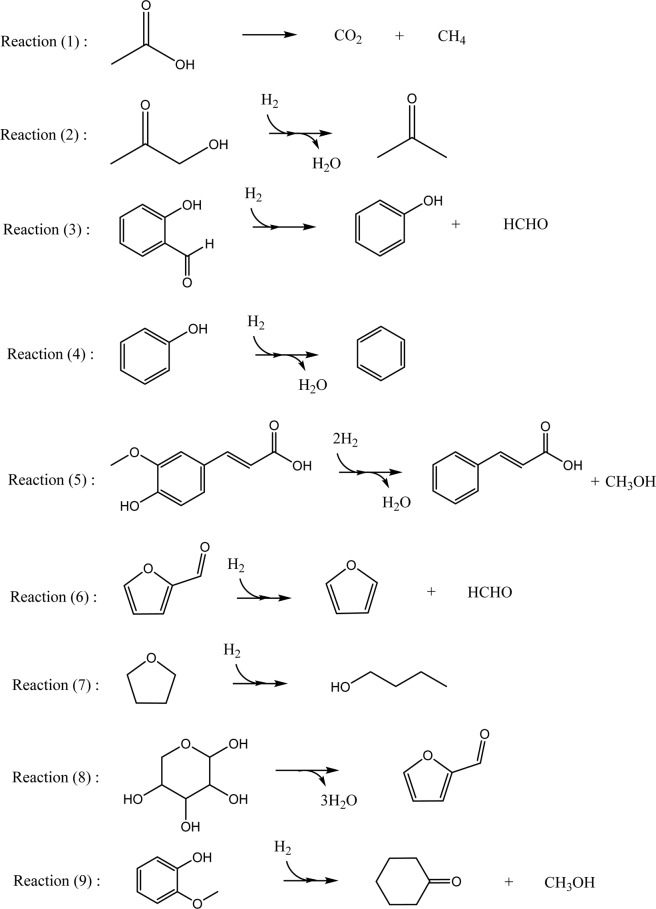
Reactions considered for this study at gas, liquid and four supercritical conditions.

Reaction scheme shown in Fig. 1 depicts all nine reactions considered in this study for studying the effect of supercritical phase on the hydrotreatment of bio-oil model compounds. In reaction (1), acetic acid is thermally degraded into carbon dioxide and methane. In reaction (2), simultaneous addition of H_2_ molecule and removal of water molecule yields acetone. Addition of an H_2_ molecule with the removal of one water molecule from salicylic acid produces phenol and formaldehyde in reaction (3). Production of benzene is then proposed via phenol in reaction (4). Addition of one H_2_ with removal of a water molecule produce benzene from phenol in reaction (4). In reaction (5), ferulic acid is converted into cinnamic acid and methanol by simultaneous hydrogenation and dehydroxylation. Removal of three water molecules from xylose produce furfural in reaction (8) whereas hydrogenation of furfural produces furan and formaldehyde in reaction (6). Also, hydrogenation of tetrahydrofuran produce butanol in reaction (7), and hydrogenation of guaiacol produce cyclohexanone and methanol in reaction (9).

## Results and Discussion

For each reaction, the effect of supercritical phase are presented at four different conditions (SC1, SC2, SC3 and SC4) and the corresponding temperature, pressure and density along with other descriptor properties of phase are given in Table 3.

### Reaction 1

In the literature^45^, it is reported that the degradation of acetic acid produces methane and CO_2_ above 450 °C in supercritical water. Below 400 °C, the compound is highly stable and requires catalysts or additives like ZrO_2_ and KOH to initiate dissociation^46^. The thermochemical parameters calculated in this study concur with this as Gibbs free energy change calculated at 373 K in the aqueous medium show the degradation to be non-spontaneous (Fig. 3). The energy change is also found to be positive showing low energy potential of the dissociation at low temperature. However, in supercritical medium particularly at conditions SC1 and SC2, the temperature and pressure become highly favourable for the decomposition to proceed. ΔG at SC1 and SC2 is found to be −9.80 kcal/mol and −8.19 kcal/mol respectively. At SC3, the free energy change is −5.01 kcal/mol. However, at SC4, despite high temperature and pressure condition, reaction (1) is found to have energy change along with near zero spontaneity. The inconsistent observation suggests that density of water is more significant parameter than temperature or pressure alone. Because in a study conducted by Matsumara *et al*.^9^, the decomposition of acetic acid into methane and CO_2_ in supercritical water is reported between 580–620 °C keeping the pressure constant; thereby keeping the density below 0.1 g/cc. However, in this study, we find that the ΔG of the process is not favourable at SC4 condition where the density is more than three times the density at SC2. Thus SC1 and SC2 conditions are more favourable for this reaction.

**Figure 2:**
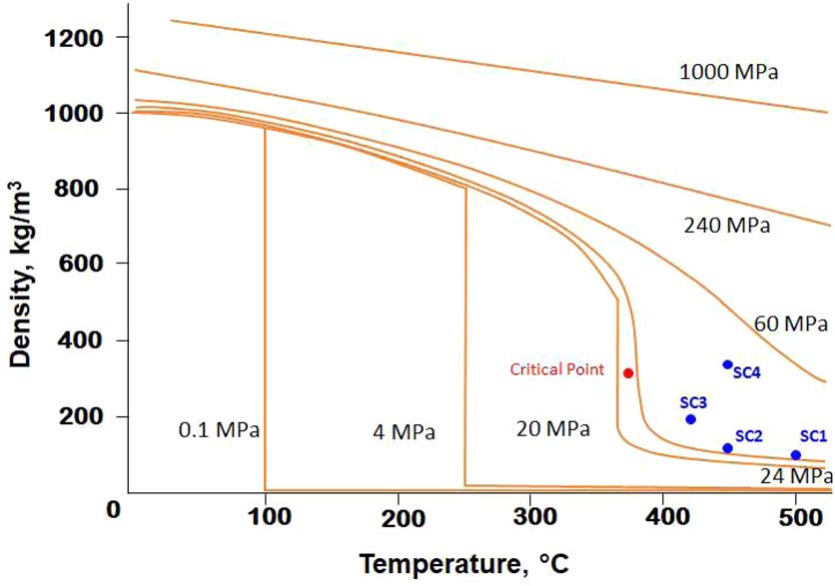
Temperature, pressure and density plot of water^55^ showing the location of four supercritical conditions – SC1, SC2, SC3 and SC4 considered in the study.

**Figure 3:**
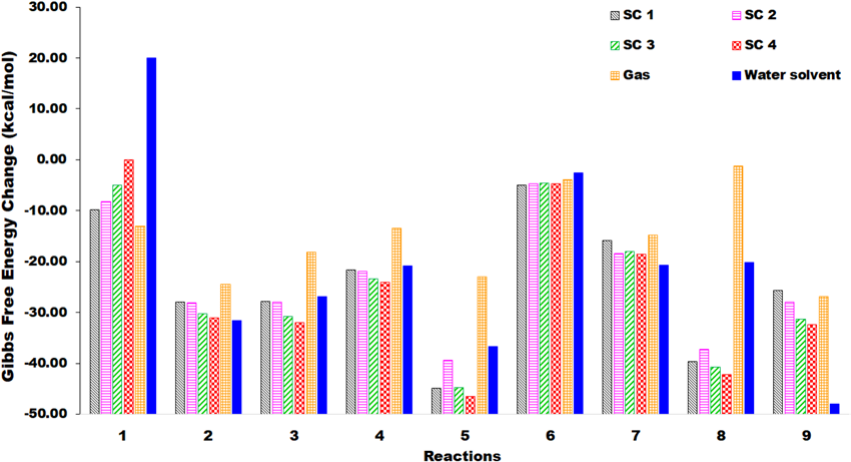
Gibbs free energy change plot of all reactions in different supercritical, gas and liquid phases.

### Reaction 2

In reaction (2), all the parameters suggest favourable reaction at all conditions. ΔG decrease from −27.96 kcal/mol at SC1 condition to −31.12 kcal/mol at SC4 condition, while still staying between the gas phase (−24.52 kcal/mol) and liquid phase (ΔG = −31.64 kcal/mol) free energy change at 423 K and 373 K respectively (Table 4). It is worth noting here that with a decrease in temperature from gas phase to liquid phase, the thermochemistry move in favourable direction. Similarly, from SC1 to SC4, as the density of medium increases, ΔG show increasing favourability. A similar finding has been reported by Kabyemela *et al*.^47^ in one of their experimental study where degradation of a similar molecule - dihydroxyacetone in sub- and supercritical water is reported to improve as the reaction shift from sub- to supercritical condition. Here, although the density of medium at SC4 is much less than the liquid water, Gibbs free energy change is almost identical to the aqueous phase ΔG. The energy change also follow a similar trend.

**Table 4:** ΔE (kcal/mol), ΔG (kcal/mol) and ΔH (kcal/mol) values of all reactions in gas, liquid and supercritical phase.

Reaction	Boiling point (K)		Gas (Temp in K)	Water Solvent (Temp in K)	SC1 (773 K,246.73 atm, ρ = 0.0897 g/cc)	SC2 (723 K,246.73 atm, ρ = 0.109 g/cc)	SC3 (700 K,300 atm, ρ = 0.190 g/cc)	SC4 (723 K,457 atm, ρ = 0.360 g/cc)
Acetic acid → CO_2_ + CH_4_	391	ΔE	0.17	11.58	7.20	7.79	10.18	10.87
		ΔG	−13.11 (423)	20.04 (373)	−9.80	−8.19	−5.01	0.05
Hydroxyacetone → acetone + water	418	ΔE	−23.50	−30.47	−24.10	−24.63	−26.87	−27.51
		ΔG	−24.52 (423)	−31.64 (373)	−27.96	−28.15	−30.27	−31.12
2-HB → Phenol	467	ΔE	−3.95	−5.44	−2.90	−3.07	−3.81	−4.00
		ΔG	−0.78 (473)	−0.52 (423)	−6.16	−6.11	−7.49	−8.00
Phenol → benzene	455	ΔE	−19.63	−25.00	−20.50	−20.86	−22.42	−22.77
		ΔG	−13.42 (473)	−20.91 (423)	−21.71	−21.92	−23.36	−24.04
Ferulic Acid → Cinnamic Acid	645	ΔE	−33.78	−47.95	−35.65	−36.63	−40.81	−42.02
		ΔG	−23.01 (673)	−36.67 (623)	−44.83	−39.43	−44.74	−46.46
Furfural → Furan	434	ΔE	−2.34	−1.37	−0.51	−0.53	−0.53	−0.47
		ΔG	−3.97 (473)	−2.61 (423)	−5.04	−4.68	−4.53	−4.66
Tetrahydrofuran → butanol	339	ΔE	−28.93	−32.03	−29.43	−29.52	−30.13	−30.32
		ΔG	−14.78 (373)	−20.65 (323)	−15.92	−18.49	−17.97	−18.57
Xylose → furfural	688	ΔE	30.91	17.43	34.66	33.06	26.27	24.38
		ΔG	−1.24 (723)	−20.23 (673)	−39.7	−37.21	−40.72	−42.19
Guaiacol → cyclohexanone	478	ΔE	−63.29	−74.09	−64.29	−64.77	−66.81	−67.43
		ΔG	−26.91 (523)	−48.02 (423)	−25.74	−27.96	−31.30	−32.35

### Reaction 3

In reaction (3), the spontaneity in the supercritical phase are found to be higher for all four supercritical conditions than in gas and liquid phase, suggesting more favourability in supercritical medium. With ΔG = −0.78 kcal/mol in gas phase at 473 K and −0.52 kcal/mol in the aqueous phase at 423 K, the reaction is barely feasible. The high energy requirement of the thermolysis of 2-hydroxybenzaldehyde in gas phase has been reported by Verma & Kishore^48^ as well. However, the spontaneity increases at SC1 condition to −6.16 kcal/mol and further reach −8.00 kcal/mol at SC4. In an experimental study, Martino & Savage^49^ have shown that the thermolysis of 2-hydrobenzaldehyde gives up to 100% yield of phenol at the supercritical conditions similar to that of this study. An interesting observation of this reaction is the slight drop in thermochemical parameters from SC1 to SC2, suggesting that the solvation effect is being offset by the temperature and pressure from SC1 to SC2. The energy change also shows the reaction to be favourable at all conditions.

### Reaction 4

The conversion of phenol to benzene is also found to be more spontaneous in the supercritical medium than gas and aqueous phase. Matsumara *et al*.^50^ have shown in their experimental work that near the critical region at low temperature and high water density, the yield of benzene increases. They reason that the reaction is stabilized due to the high dielectric constant, which is consistent with our hypothesis. The energy change, although less favourable than gas and aqueous phase, are largely negative at all supercritical density exhibiting exothermic reaction conditions.

### Reaction 5

The conversion of ferulic acid to cinnamic acid show a mixed trend in thermochemical parameters with increasing supercritical water densities. Although the reaction is most spontaneous in supercritical conditions, the free energy change is found to lie between that of gas-phase and aqueous phase. The gas-phase study has also been conducted by Verma *et al*.^51^ at 598 K and above where they report a decrease of less than 3 kcal/mol in ΔG for every 100 K increase in temperature. The introduction of solvation model also shifts ΔG in favourable direction, as seen in this study. Also, the gas phase and aqueous phase comparisons are made at 673 K and 623 K respectively, which is very close to the critical temperature. Thus the anomalous behaviour of the ΔG may be explained as the offsetting of the solvation effect by only the pressure effect. The best condition based on ΔG for this reaction is found to be at SC4.

### Reaction 6

In the conversion of furfural to furan, ΔG for gas and liquid phase did not show much spontaneity at any condition and any temperature. The variations between the spontaneity for the reaction from SC1 to SC 4 is also not more than 0.51 kcal/mol. In addition, the free energy change and the energy change lie within 5 kcal/mol of 0 kcal/mol point for all medium within the present range of temperature, pressure and density. This suggests that the reaction is not going to proceed to completion even in the supercritical medium. The thermochemistry is not only found to be unfavourable, but is not much affected by the change in operating parameters in any medium. Thus catalytic reactions, like the ones patented in the work of Binder & Wang^52^, is recommended for this reaction.

### Reaction 7

In reaction (7), ΔG varies from −15.92 kcal/mol at SC1 condition to −18.57 kcal/mol at SC4 condition, nevertheless staying between gas phase (ΔG = −14.78 kcal/mol) and liquid phase (ΔG = −20.65 kcal/mol) free energy change. However, it is interesting to observe that the spontaneity rise from SC1 to SC2, only to fall at SC3 and rise again at SC4. Similar to reaction (3) and (5), this decrease in ΔG at SC2 may have been caused due to offset of effect of solvation by the change in temperature-pressure which resulted in net increase in spontaneity. However, it has been reported by Agrawal *et al*.^53^ that the increase in temperature does not influence the spontaneity of the conversion in a significant way. Thus from SC1 to SC2, since the pressure is constant and temperature effect can be neglected, there is only solvation effect at play. The solvation effect shifts the reaction towards better spontaneity. However, from SC2 to SC3, there is an increase in pressure of 53.27 atm, which offsets the positive solvation effect and cause the free energy change to increase. Thus, it can be inferred that increase in pressure acts as a deterrent to the free energy change for this reaction. The energy change is not much influenced by either the medium or by operating conditions since the deviation between them is less than 4 kcal/mol. Based on the thermochemistry, it can be recommended that the conversion be conducted at SC2 conditions.

### Reaction 8

Xylose to furfural conversion is found to be fairly spontaneous in supercritical conditions. While free energy change in the gas phase is −1.24 kcal/mol, it is found to be −39.70 kcal/mol at SC1 condition. At SC2 condition, ΔG increase to −37.21 kcal/mol and subsequently decrease to −42.19 kcal/mol at SC4 condition. At SC1, it is found to be 24.14 kcal/mol and decrease to 14.24 kcal/mol at SC4 condition. However, in a different study conducted by same authors^53^, they found M06-2X and B3LYP functional to miserably fail in predicting free energy change of this reaction with the solvent model.

### Reaction 9

In reaction (9), it is observed that the free energy change at SC1 is less than that of the gas phase and liquid phase. ΔG at SC1 is found to be −25.74 whereas in gas and liquid phase, they are calculated as −26.91 kcal/mol and −48.02 kcal/mol respectively. The effect of solvation is very prominent in this reaction as evident from the significantly smaller ΔG in aqueous phase than gas phase. The effect of temperature can be seen in the works of Verma & Kishore^54^ who report a decrease in spontaneity with an increase in temperature in the gas phase. However, free energy change at SC1 is almost similar to ΔG of gas-phase despite large temperature and pressure difference. Further, as the operating conditions are made more extreme at SC4, the spontaneity increase instead of decreasing. This shows that in this particular reaction, the solvation effect is dominant over the temperature-pressure parameters. However, the electronic energy change is found to lie between gas and aqueous phase energy change.

### Effect of molecular structure

The Gibbs free energy change in all the reactions are found to be more favourable in liquid phase than in the gas phase, except in the case of reaction 1 and reaction 6. In reaction 1, the products are primarily gaseous and not very soluble in the water medium when compared to the reactant. Thus as the density shifts towards water from sc1 to sc4, the ΔG increases showing unfavourable trend. It is also observed that reactions like 3 and 6, which involve direct cleavage of non-alcoholic group show little to no advantage in the supercritical phase; whereas reactions where the product contain -OH group and/or water are found to be more favourable in supercritical phase, such as in reaction 2, 4, 5 and 8. Such reactions also become more favourable with increase in density from sc1 to sc4. This could be due to better solubility, and thus stability of the alcoholic products in the medium. Another noteworthy observation of the study is that unlike gas and liquid phase, the heavier molecules like ferulic acid and xylose in reaction 5 and 8 show more favourable energy in supercritical phase than in the gas and liquid phase. Other compounds do not show any influence of molecular weight on the thermodynamics of the reaction.

Gibbs free energy change of all the reactions are calculated in the gas phase, liquid phase and at four different supercritical water density (Fig. 3). For reactions (1), (2) and (7), the spontaneity at all four supercritical water density is found to lie between the spontaneity of gas phase and liquid phase of the respective reactions. In reaction (3) and (4), the spontaneity in SC phase is found to be higher for all four SC water density than in gas and liquid phase, suggesting better thermochemistry at SC conditions. Reaction (5), (6) and (8) did not show any trend in ΔG with increasing SC water densities. Although, they are also found to be more spontaneous in SC phase than gas and liquid phase. Finally, in reaction (9), it is observed that the free energy change at SC1 is less than that of gas phase and liquid phase.

## Conclusions

The descriptors of the SMD model required for supercritical phase were defined manually at the supercritical water conditions and the system was validated with reported literature results. The validated system was then used to study a set of nine reactions. The nine reactions were chosen such that they covered variation in molecular weight, functional groups, types of bond and aromatic and linear compounds.

The conversion of aromatic compounds to less oxy-functional compound like in reaction (3), (4) and (5), was found to be more favourable in the supercritical phase than gas or liquid phase. The ΔG of these reactions was more favourable in supercritical phase than gas or liquid medium. But it was observed that the conversion of smaller and simpler compounds, like in reaction (1), (2) and (7), were not more favourable in supercritical phase than gas phase and liquid phase. The spontaneity of reaction (1), (2) and (7) in the supercritical phase was less than the spontaneity in liquid water. In several reactions, the effect of high temperature and pressure condition displayed an offsetting effect on the solvation parameters of the supercritical phase.

The study shows that supercritical water can be a good medium for heavy aromatic compound degradation and upgrading. Since the major fraction of the lignocellulosic biomass is phenolic compounds, supercritical water can be a good medium for its upgrading. The present study also shows that conditions at higher density of water (like sc4 condition) in supercritical phase may be more favourable for such reactions. However, the lighter molecules such as acetic acid, may show a reverse trend. Hence, the readers should keep this offset in consideration when choosing the parameters.

## Supplementary information

Supplementary information.
