# Supplementary material for: Study of Conversion of Bio-oil Model Compounds in Supercritical Water Using Density Functional Theory
Source: Sci Rep. 2020 Jun 8;10:9247. doi: 10.1038/s41598-020-66237-w (PMC7280221; doi:10.1038/s41598-020-66237-w)
Supplement: Supplementary file 1 — Supplementary information. [file 41598_2020_66237_MOESM1_ESM.pdf]

Study of Conversion of Bio-oil Model Compounds in Supercritical Water Using Density Functional Theory

by

Kushagra Agrawal and **Nanda Kishore***

Department of Chemical Engineering, Indian Institute of Technology Guwahati, Guwahati, Assam, India – 781039

*Corresponding Author

Nanda Kishore

Department of Chemical Engineering

Indian Institute of Technology Guwahati

Guwahati, Assam, India – 781039

Email ID: nkishore@iitg.ac.in

Table 1S: Optimized geometrical coordinate of all the reactants and products in gas, water and four supercritical conditions.

Reaction Number	Common name	Phase	Coordinate			
1	Acetic acid	Gas	Symbol	X	Y	Z
			C	-1.38888	-0.1236	0.000013
			H	-1.65513	-0.70733	0.879919
			H	-1.91355	0.825301	0.000106
			H	-1.65544	-0.70755	-0.87963
			C	0.087746	0.126306	-0.00011
			O	0.788566	-1.0282	-3.7E-05
			H	1.724258	-0.79137	0.000446
			O	0.624765	1.19879	0.000007
		Liquid	Symbol	X	Y	Z
			C	-1.38609	-0.1342	0.000575
			H	-1.64415	-0.76941	0.846938
			H	-1.92043	0.808128	0.055129
			H	-1.653	-0.66621	-0.91254
			C	0.08198	0.116857	0.00168
			O	0.800672	-1.01716	0.000356
			H	1.743591	-0.7927	-0.00423
			O	0.61166	1.207687	-0.00021
		SC1	Symbol	X	Y	Z
			C	-1.38671	-0.12623	-0.00037
			H	-1.65507	-0.68033	0.89897
			H	-1.91703	0.819764	-0.03367
			H	-1.64848	-0.74262	-0.85978
			C	0.085194	0.126745	-0.00089
			O	0.790344	-1.027	-0.00016
			H	1.729572	-0.79955	0.002121
			O	0.62217	1.201956	0.000152
		SC2	Symbol	X	Y	Z
			C	-1.38661	-0.12659	-0.00034
			H	-1.65454	-0.68241	0.898104
			H	-1.91714	0.819389	-0.03173
			H	-1.64815	-0.74108	-0.86116
			C	0.084925	0.125803	-0.00087
			O	0.790709	-1.02617	-0.00015
			H	1.730749	-0.80054	0.002072
			O	0.621693	1.202339	0.000153
		SC3	Symbol	X	Y	Z
			C	-1.38626	-0.12695	-2.2E-05
			H	-1.65015	-0.71081	0.881789
			H	-1.9177	0.819148	-0.00173
			H	-1.64984	-0.71406	-0.87972
			C	0.083983	0.122201	-0.00003
			O	0.791131	-1.02326	-2.8E-05

			H	1.734752	-0.80643	0.000217
			O	0.620943	1.203342	-2E-06
		SC4	Symbol	X	Y	Z
			C	-1.38611	-0.12716	0.000336
			H	-1.64678	-0.74702	0.857921
			H	-1.91775	0.818108	0.037475
			H	-1.65283	-0.67721	-0.90249
			C	0.083785	0.120995	0.001196
			O	0.791376	-1.02241	0.000349
			H	1.735876	-0.80767	-0.00356
			O	0.620556	1.203756	-0.00017
	Methane	Gas	Symbol	X	Y	Z
			C	0	0	0
			H	0.627634	0.627634	0.627634
			H	-0.62763	-0.62763	0.627634
			H	-0.62763	0.627634	-0.62763
			H	0.627634	-0.62763	-0.62763
		Liquid	Symbol	X	Y	Z
			C	0	0	0
			H	0.628219	0.628219	0.628219
			H	-0.62822	-0.62822	0.628219
			H	-0.62822	0.628219	-0.62822
			H	0.628219	-0.62822	-0.62822
		SC1	Symbol	X	Y	Z
			C	0	0	0
			H	0.628197	0.628197	0.628197
			H	-0.6282	-0.6282	0.628197
			H	-0.6282	0.628197	-0.6282
			H	0.628197	-0.6282	-0.6282
		SC2	Symbol	X	Y	Z
			C	0	0	0
			H	0.628238	0.628238	0.628238
			H	-0.62824	-0.62824	0.628238
			H	-0.62824	0.628238	-0.62824
			H	0.628238	-0.62824	-0.62824
		SC3	Symbol	X	Y	Z
			C	0	0	0
			H	0.628403	0.628403	0.628403
			H	-0.6284	-0.6284	0.628403
			H	-0.6284	0.628403	-0.6284
			H	0.628403	-0.6284	-0.6284
		SC4	Symbol	X	Y	Z
			C	0	0	0
			H	0.628456	0.628456	0.628456
			H	-0.62846	-0.62846	0.628456
			H	-0.62846	0.628456	-0.62846
			H	0.628456	-0.62846	-0.62846

	Carbon dioxide	Gas	Symbol	X	Y	Z
			C	0	0	0
			O	0	0	1.154334
			O	0	0	-1.15433
		Liquid	Symbol	X	Y	Z
			C	0	0	0
			O	0	0	1.154668
			O	0	0	-1.15467
		SC1	Symbol	X	Y	Z
			C	0	0	0
			O	0	0	1.154203
			O	0	0	-1.1542
		SC2	Symbol	X	Y	Z
			C	0	0	0
			O	0	0	1.154205
			O	0	0	-1.15421
		SC3	Symbol	X	Y	Z
			C	0	0	0
			O	0	0	1.154218
			O	0	0	-1.15422
		SC4	Symbol	X	Y	Z
			C	0	0	0
			O	0	0	1.154219
			O	0	0	-1.15422
2	acetol	Gas	Symbol	X	Y	Z
			C	0.718595	-0.77243	0.04091
			H	0.619569	-1.47978	-0.78873
			H	0.664031	-1.35133	0.970458
			C	-0.47133	0.16231	0.01842
			C	-1.83724	-0.46174	-0.02086
			H	-1.85661	-1.41189	0.511558
			H	-2.56764	0.227384	0.393913
			H	-2.09227	-0.65551	-1.06488
			O	1.926691	-0.07684	-0.04603
			H	1.703605	0.862783	-0.01682
			O	-0.29305	1.356781	0.016491
		Liquid	Symbol	X	Y	Z
			C	0.70144	-0.77724	0.044057
			H	0.600964	-1.48485	-0.78149
			H	0.645445	-1.34299	0.978533
			C	-0.47465	0.159786	0.017991
			C	-1.83092	-0.4572	-0.02127
			H	-1.84879	-1.39113	0.538497
			H	-2.57412	0.238976	0.358695
			H	-2.05642	-0.68791	-1.06557
			O	1.928577	-0.08195	-0.05139
			H	1.717648	0.860049	0.015012
			O	-0.28607	1.363921	0.015344

		SC1	Symbol	X	Y	Z
			C	0.713673	-0.77664	0.03644
			H	0.623442	-1.47778	-0.79915
			H	0.663109	-1.36048	0.962318
			C	-0.47231	0.158185	0.016936
			C	-1.83864	-0.45533	-0.01819
			H	-1.87088	-1.39206	0.537887
			H	-2.5682	0.249337	0.37339
			H	-2.08698	-0.6752	-1.05944
			O	1.927513	-0.07507	-0.04129
			H	1.707232	0.866182	-0.01384
			O	-0.28802	1.354158	0.014754
		SC2	Symbol	X	Y	Z
			C	0.712606	-0.77673	0.036963
			H	0.62198	-1.47825	-0.798
			H	0.66187	-1.35914	0.963533
			C	-0.47262	0.158192	0.016964
			C	-1.83812	-0.45535	-0.01838
			H	-1.86937	-1.39175	0.538293
			H	-2.56866	0.249019	0.37198
			H	-2.08463	-0.67628	-1.05993
			O	1.927617	-0.07562	-0.04196
			H	1.709028	0.866096	-0.01207
			O	-0.2878	1.354816	0.014825
		SC3	Symbol	X	Y	Z
			C	0.708214	-0.77707	0.039383
			H	0.615503	-1.48071	-0.79244
			H	0.656352	-1.35296	0.969176
			C	-0.47376	0.157956	0.016925
			C	-1.83591	-0.45543	-0.01917
			H	-1.8625	-1.39126	0.538404
			H	-2.57012	0.246973	0.368292
			H	-2.07587	-0.67894	-1.06203
			O	1.928133	-0.0778	-0.04546
			H	1.717384	0.865524	0.000713
			O	-0.28714	1.357623	0.014843
		SC4	Symbol	X	Y	Z
			C	0.706928	-0.77699	0.041064
			H	0.6133	-1.48319	-0.78813
			H	0.654562	-1.34897	0.973125
			C	-0.47405	0.157974	0.01705
			C	-1.83501	-0.45559	-0.01992
			H	-1.85998	-1.39248	0.535945
			H	-2.5706	0.245195	0.368057
			H	-2.0728	-0.67819	-1.06359
			O	1.928106	-0.07856	-0.04767
			H	1.72007	0.865114	0.005739
			O	-0.28708	1.358587	0.015127

	Acetone	Gas	Symbol	X	Y	Z
			C	-1.28253	-0.61172	-0.00264
			H	-1.38666	-1.12253	0.956763
			H	-1.25353	-1.37887	-0.77735
			C	0.000003	0.185112	0.000005
			C	1.282543	-0.6117	0.00264
			H	1.253549	-1.37888	0.77733
			H	1.386665	-1.12249	-0.95677
			H	2.128254	0.052066	0.155987
			O	-1.2E-05	1.391058	-1E-06
			H	-2.12825	0.052049	-0.15598
		Liquid	Symbol	X	Y	Z
			C	-1.27464	-0.60965	-0.00369
			H	-1.39065	-1.0727	0.9792
			H	-1.21525	-1.41534	-0.73577
			C	0.000001	0.17467	0.000001
			C	1.274641	-0.60964	0.003687
			H	1.215258	-1.41533	0.735773
			H	1.39065	-1.07269	-0.9792
			H	2.12456	0.036214	0.206804
			O	-5E-06	1.396416	0
			H	-2.12456	0.036202	-0.20681
		SC1	Symbol	X	Y	Z
			C	-1.28189	-0.60972	-0.00289
			H	-1.39596	-1.1073	0.96307
			H	-1.25127	-1.38822	-0.76681
			C	-1E-06	0.180307	0.000007
			C	1.281853	-0.60977	0.002896
			H	1.251121	-1.38843	0.766644
			H	1.39596	-1.10717	-0.96314
			H	2.12599	0.053688	0.170766
			O	0.000042	1.389839	0
			H	-2.12598	0.053829	-0.17061
		SC2	Symbol	X	Y	Z
			C	-1.28138	-0.6096	-0.00294
			H	-1.39528	-1.1055	0.963969
			H	-1.24925	-1.38945	-0.76542
			C	0	0.179617	0
			C	1.281374	-0.60962	0.002941
			H	1.249214	-1.38949	0.765386
			H	1.395275	-1.10548	-0.96398
			H	2.12588	0.053082	0.172293
			O	0.000011	1.390163	0.000001
			H	-2.12587	0.053123	-0.17227
		SC3	Symbol	X	Y	Z
			C	-1.27938	-0.60898	-0.00311
			H	-1.39086	-1.10003	0.966857
			H	-1.2411	-1.39318	-0.76082

			C	-6E-06	0.176467	0.000058
			C	1.279398	-0.60894	0.003127
			H	1.240593	-1.39437	0.759539
			H	1.391587	-1.09857	-0.96749
			H	2.125875	0.049967	0.178773
			O	-0.00002	1.391844	-1.8E-05
			H	-2.12602	0.050154	-0.17718
		SC4	Symbol	X	Y	Z
			C	-1.27873	-0.60881	-0.00313
			H	-1.38951	-1.09856	0.967571
			H	-1.23876	-1.39413	-0.7595
			C	0.000001	0.17564	0.000001
			C	1.278721	-0.60882	0.003133
			H	1.238753	-1.39413	0.759497
			H	1.389507	-1.09856	-0.96757
			H	2.126046	0.049158	0.178537
			O	0.000004	1.392373	0
			H	-2.12605	0.049167	-0.17854
	Water	Gas	Symbol	X	Y	Z
			O	0	0	0.11589
			H	0	0.761986	-0.46356
			H	0	-0.76199	-0.46356
		Liquid	Symbol	X	Y	Z
			O	0	0	0.117649
			H	0	0.760322	-0.4706
			H	0	-0.76032	-0.4706
		SC1	Symbol	X	Y	Z
			O	0	0	0.115895
			H	0	0.763322	-0.46358
			H	0	-0.76332	-0.46358
		SC2	Symbol	X	Y	Z
			O	0	0	0.115999
			H	0	0.76336	-0.464
			H	0	-0.76336	-0.464
		SC3	Symbol	X	Y	Z
			O	0	0	0.116538
			H	0	0.763215	-0.46615
			H	0	-0.76322	-0.46615
		SC4	Symbol	X	Y	Z
			O	0	0	0.11671
			H	0	0.763094	-0.46684
			H	0	-0.76309	-0.46684
3	2-Hydroxy benzene	Gas	Symbol	X	Y	Z
			C	1.753183	0.723946	-4.6E-05
			C	0.382999	0.978515	-5E-06
			C	-0.52524	-0.08842	0.000035
			C	-0.0342	-1.40117	0.000052
			C	1.320424	-1.65449	0.000026

			C	2.212493	-0.57898	-3.4E-05
			H	2.429942	1.566221	-7.9E-05
			H	-0.75984	-2.20278	0.000077
			H	1.688613	-2.66995	0.000041
			H	3.278302	-0.76269	-6.5E-05
			C	-1.98469	0.108018	-9E-06
			H	-2.3575	1.149309	-1.7E-05
			O	-2.78918	-0.79006	-5.5E-05
			O	0.026153	2.289612	0.000032
			H	-0.92518	2.398915	0.000117
		Liquid	Symbol	X	Y	Z
			C	1.750152	0.725465	-0.01062
			C	0.378931	0.978966	0.000114
			C	-0.52932	-0.09138	-7.6E-05
			C	-0.03138	-1.40406	0.010557
			C	1.32392	-1.65471	0.00712
			C	2.214213	-0.57661	-0.00852
			H	2.431058	1.565271	-0.01188
			H	-0.743	-2.21814	0.012325
			H	1.694263	-2.66961	0.012179
			H	3.28062	-0.75707	-0.01403
			C	-1.98038	0.112187	-0.04379
			H	-2.34996	1.138304	-0.15914
			O	-2.79054	-0.79717	0.022601
			O	0.02157	2.294495	0.009831
			H	-0.91802	2.423441	0.172395
		SC1	Symbol	X	Y	Z
			C	1.753405	0.7192	-0.00513
			C	0.384246	0.976352	0.000817
			C	-0.52719	-0.08732	0.002364
			C	-0.03857	-1.40151	0.006427
			C	1.315141	-1.65749	0.003251
			C	2.209929	-0.58452	-0.00489
			H	2.433237	1.559759	-0.00755
			H	-0.7639	-2.20419	0.008877
			H	1.681055	-2.67425	0.005166
			H	3.275708	-0.77069	-0.00912
			C	-1.9834	0.112768	-0.0143
			H	-2.35785	1.150227	-0.05344
			O	-2.78856	-0.78875	0.003976
			O	0.034089	2.295059	0.003194
			H	-0.91384	2.423771	0.067472
		SC2	Symbol	X	Y	Z
			C	1.753536	0.718216	-0.00505
			C	0.384389	0.976525	0.000894
			C	-0.52774	-0.08697	0.002401
			C	-0.03937	-1.40154	0.006394
			C	1.31419	-1.65838	0.003188

			C	2.209577	-0.58573	-0.0049
			H	2.434289	1.558143	-0.00746
			H	-0.76434	-2.20455	0.008754
			H	1.679515	-2.6754	0.004992
			H	3.275317	-0.77236	-0.00921
			C	-1.98329	0.113929	-0.01452
			H	-2.35741	1.150615	-0.05414
			O	-2.78914	-0.7883	0.004218
			O	0.036192	2.295601	0.003306
			H	-0.91158	2.428724	0.06643
		SC3	Symbol	X	Y	Z
			C	1.75354	0.715872	-0.00648
			C	0.384096	0.977489	0.001352
			C	-0.52974	-0.08632	0.003156
			C	-0.04112	-1.40192	0.008175
			C	1.312131	-1.66088	0.004051
			C	2.208901	-0.58862	-0.0063
			H	2.437434	1.553675	-0.00971
			H	-0.76328	-2.20753	0.010906
			H	1.67621	-2.67853	0.006214
			H	3.274617	-0.77608	-0.012
			C	-1.98274	0.117824	-0.01927
			H	-2.355	1.150949	-0.07121
			O	-2.79088	-0.78804	0.005961
			O	0.041422	2.296969	0.004522
			H	-0.90468	2.445374	0.083812
		SC4	Symbol	X	Y	Z
			C	1.753462	0.715578	-0.00737
			C	0.383864	0.977865	0.001332
			C	-0.53024	-0.08622	0.003325
			C	-0.04127	-1.402	0.008983
			C	1.311959	-1.6613	0.00461
			C	2.208894	-0.58896	-0.00691
			H	2.438132	1.552863	-0.01101
			H	-0.76233	-2.20861	0.011999
			H	1.675915	-2.67904	0.007206
			H	3.274635	-0.77644	-0.01307
			C	-1.98259	0.118711	-0.02114
			H	-2.3545	1.150851	-0.07776
			O	-2.7912	-0.78837	0.006491
			O	0.042097	2.297106	0.005343
			H	-0.90353	2.44844	0.090949
4	Benzene	Gas	Symbol	X	Y	Z
			C	-1.38511	-0.11768	0
			C	-0.59063	-1.25829	0.000009
			C	0.794474	-1.14064	-4E-06
			C	1.385111	0.117676	0.000002
			C	0.59063	1.258293	0.000004

			C	-0.79447	1.140636	-6E-06
			H	-2.46274	-0.20932	0.000001
			H	-1.05009	-2.23738	-6E-06
			H	1.412488	-2.02819	-1.1E-05
			H	2.462745	0.209317	-9E-06
			H	1.05009	2.237379	0.000011
			H	-1.41249	2.02819	-9E-06
		Liquid	Symbol	X	Y	Z
			C	1.22671	-0.65743	-1E-06
			C	1.182724	0.733614	0.000025
			C	-0.04399	1.390935	-1.3E-05
			C	-1.22671	0.657429	0.000006
			C	-1.18272	-0.73362	0.000011
			C	0.043989	-1.39094	-1.7E-05
			H	2.180095	-1.16851	0.000004
			H	2.101924	1.304008	-0.00002
			H	-0.07826	2.47212	-3.5E-05
			H	-2.1801	1.1685	-2.4E-05
			H	-2.10193	-1.304	0.000032
			H	0.078267	-2.47212	-2.8E-05
		SC1	Symbol	X	Y	Z
			C	0.022085	-1.38966	-1E-06
			C	1.21469	-0.67578	0.000017
			C	1.192521	0.714006	-9E-06
			C	-0.02208	1.389662	0.000004
			C	-1.21469	0.675775	0.000008
			C	-1.19252	-0.71401	-1.1E-05
			H	0.039043	-2.47149	0.000002
			H	2.160235	-1.20172	-1.4E-05
			H	2.12074	1.26983	-2.3E-05
			H	-0.03905	2.471491	-1.7E-05
			H	-2.16023	1.201722	0.000022
			H	-2.12074	-1.26983	-1.8E-05
		SC2	Symbol	X	Y	Z
			C	-1.38844	-0.06961	-1E-06
			C	-0.63391	-1.2371	0.000028
			C	0.754472	-1.16745	-1.4E-05
			C	1.388435	0.069606	0.000006
			C	0.633906	1.237097	0.000013
			C	-0.75447	1.167453	-1.9E-05
			H	-2.4691	-0.12387	0.000004
			H	-1.12694	-2.20025	-2.3E-05
			H	1.341715	-2.07618	-3.8E-05
			H	2.469102	0.123873	-2.8E-05
			H	1.126945	2.200248	0.000037
			H	-1.34172	2.076175	-3.1E-05
		SC3	Symbol	X	Y	Z
			C	1.027072	-0.93791	-1E-06

			C	1.325792	0.420478	0.000025
			C	0.29872	1.358423	-1.3E-05
			C	-1.02707	0.937907	0.000006
			C	-1.32579	-0.42048	0.000011
			C	-0.29872	-1.35842	-1.7E-05
			H	1.826178	-1.66775	0.000004
			H	2.357351	0.747509	-0.00002
			H	0.531481	2.415324	-3.4E-05
			H	-1.82618	1.66774	-2.5E-05
			H	-2.35735	-0.7475	0.000033
			H	-0.53148	-2.41533	-2.7E-05
		SC4	Symbol	X	Y	Z
			C	0.78621	-1.14762	-1E-06
			C	1.386986	0.107036	0.000025
			C	0.600775	1.254692	-1.3E-05
			C	-0.78621	1.147616	0.000006
			C	-1.38699	-0.10704	0.000011
			C	-0.60078	-1.25469	-1.7E-05
			H	1.397827	-2.04052	0.000004
			H	2.465996	0.190187	-2.1E-05
			H	1.068459	2.230704	-3.5E-05
			H	-1.39783	2.040512	-2.5E-05
			H	-2.466	-0.19018	0.000033
			H	-1.06845	-2.23071	-2.8E-05
3	formaldehyde	Gas	Symbol	X	Y	Z
			C	0	0.526482	0
			H	0.937092	1.10531	0
			H	-0.9371	1.105303	0
			O	0	-0.67119	0
		Liquid	Symbol	X	Y	Z
			C	0	0.533743	0
			H	0.936751	1.103307	0
			H	-0.93676	1.103297	0
			O	0	-0.67613	0
		SC1	Symbol	X	Y	Z
			C	0.000001	0.527969	0
			H	0.937824	1.104714	0
			H	-0.93783	1.104698	0
			O	0.000001	-0.67215	0
		SC2	Symbol	X	Y	Z
			C	0.000001	0.528497	0
			H	0.93782	1.104459	0
			H	-0.93783	1.104444	0
			O	0.000001	-0.67249	0
		SC3	Symbol	X	Y	Z
			C	0	0.530814	0
			H	0.937691	1.103617	0
			H	-0.9377	1.103603	0

			O	0	-0.67401	0
		SC4	Symbol	X	Y	Z
			C	0	0.531458	0
			H	0.937642	1.103465	0
			H	-0.93765	1.103451	0
			O	0	-0.67446	0
4	Phenol	Gas	Symbol	X	Y	Z
			C	-1.84833	0.029188	-1.2E-05
			C	-1.1252	1.21406	-0.00001
			C	0.264612	1.193009	0.000017
			C	0.933327	-0.02585	0.000034
			C	0.219087	-1.21879	0.000008
			C	-1.16691	-1.18414	0.000002
			H	-2.92865	0.049027	-3.1E-05
			H	-1.64033	2.164891	-2.5E-05
			H	0.826639	2.119429	0.000055
			H	0.763974	-2.15205	0.000009
			H	-1.71949	-2.11379	0.000003
			O	2.295441	-0.11033	-2.7E-05
			H	2.674849	0.770255	-3.5E-05
		Liquid	Symbol	X	Y	Z
			C	-1.85003	0.035342	0.000001
			C	-1.12027	1.219305	-6E-06
			C	0.270166	1.194089	-7E-06
			C	0.929283	-0.03055	0.000036
			C	0.213152	-1.22267	-6E-06
			C	-1.17533	-1.18253	-1E-06
			H	-2.93078	0.06037	0.000009
			H	-1.63206	2.172241	-1.4E-05
			H	0.845233	2.111655	0.000009
			H	0.748648	-2.16242	-6E-06
			H	-1.73188	-2.11011	0.000001
			O	2.301968	-0.11499	-1.8E-05
			H	2.683255	0.770179	0.000045
		SC1	Symbol	X	Y	Z
			C	-1.84852	0.02845	0
			C	-1.12605	1.213749	0.000001
			C	0.263736	1.194354	0
			C	0.93036	-0.02462	-1E-06
			C	0.218809	-1.21828	-1E-06
			C	-1.16723	-1.18474	0
			H	-2.92943	0.048024	-1E-06
			H	-1.64235	2.164496	0.000002
			H	0.825774	2.120798	0.000001
			H	0.765367	-2.15132	-3E-06
			H	-1.71954	-2.11511	-1E-06
			O	2.298651	-0.11106	0.000006
			H	2.684297	0.768117	-3.4E-05

		SC2	Symbol	X	Y	Z
			C	-1.84863	0.029059	0
			C	-1.12552	1.214244	0.000001
			C	0.264349	1.194325	0
			C	0.930278	-0.02514	-1E-06
			C	0.218333	-1.21867	-1E-06
			C	-1.16791	-1.18457	0
			H	-2.9296	0.049114	-1E-06
			H	-1.64146	2.165241	0.000002
			H	0.827393	2.120125	0.000003
			H	0.764149	-2.15226	-4E-06
			H	-1.72062	-2.11476	-1E-06
			O	2.298764	-0.11139	0.000007
			H	2.684608	0.768134	-4.5E-05
		SC3	Symbol	X	Y	Z
			C	-1.84915	0.031336	0.000021
			C	-1.1235	1.216281	-5E-06
			C	0.266751	1.194606	-3.2E-05
			C	0.929924	-0.02694	0.000048
			C	0.21658	-1.22017	-1.1E-05
			C	-1.17063	-1.18405	0.000009
			H	-2.93037	0.053194	0.000032
			H	-1.63812	2.168216	-5.4E-05
			H	0.834492	2.117403	-0.00017
			H	0.759148	-2.15613	0.000023
			H	-1.72477	-2.11361	0.000033
			O	2.299151	-0.11291	-0.00013
			H	2.686592	0.76781	0.000946
		SC4	Symbol	X	Y	Z
			C	-1.84933	0.031852	0.000012
			C	-1.12304	1.216797	-3E-06
			C	0.267326	1.194802	-2.1E-05
			C	0.929878	-0.02728	0.000054
			C	0.21617	-1.2205	-7E-06
			C	-1.17133	-1.18397	0.000007
			H	-2.93062	0.054132	0.000012
			H	-1.63734	2.168963	-4.9E-05
			H	0.836398	2.116746	-0.00013
			H	0.757777	-2.15715	0.000023
			H	-1.72575	-2.11342	0.000023
			O	2.299243	-0.11337	-0.00011
			H	2.687533	0.767521	0.000729
5	Cinnamic acid	Gas	Symbol	X	Y	Z
			C	-3.62932	-0.24289	0.000007
			C	-3.1254	1.050567	-4.4E-05
			C	-1.75268	1.257773	-5.3E-05
			C	-0.86532	0.179052	-1.1E-05
			C	-1.38607	-1.11956	0.00004

			C	-2.75497	-1.32696	0.000049
			H	-1.35708	2.26527	-9.3E-05
			H	-0.71991	-1.97079	0.000071
			H	-3.14517	-2.33533	0.000088
			C	0.570889	0.460542	-2.2E-05
			H	0.85459	1.507964	-7.2E-05
			C	1.566882	-0.42916	0.000023
			H	1.421869	-1.4992	0.000076
			C	2.958158	0.052783	0.000002
			O	3.837155	-0.97285	0.000055
			H	4.720611	-0.58498	0.000038
			O	3.316375	1.202474	-5.3E-05
			H	-3.79847	1.896587	-7.6E-05
			H	-4.69768	-0.40936	0.000015
		Liquid	Symbol	X	Y	Z
			C	-3.62431	-0.2579	0.000005
			C	-3.13314	1.042149	-5.3E-05
			C	-1.76154	1.263718	-5.9E-05
			C	-0.86583	0.189942	-0.00001
			C	-1.37138	-1.11648	0.000046
			C	-2.73957	-1.33541	0.000054
			H	-1.37426	2.274618	-0.0001
			H	-0.69774	-1.96227	0.000085
			H	-3.1205	-2.34753	0.000098
			C	0.567	0.485439	-1.6E-05
			H	0.828053	1.53836	-5.1E-05
			C	1.561354	-0.40984	0.000016
			H	1.401424	-1.47814	0.00005
			C	2.9542	0.045237	0.000005
			O	3.818004	-0.98529	0.000037
			H	4.721104	-0.63455	0.000027
			O	3.335413	1.200486	-0.00003
			H	-3.81484	1.881579	-9.2E-05
			H	-4.69132	-0.43475	0.000011
		SC1	Symbol	X	Y	Z
			C	-3.62617	-0.24671	0.025657
			C	-3.12461	1.046679	0.074183
			C	-1.75309	1.258322	0.045513
			C	-0.86471	0.182487	-0.02388
			C	-1.38292	-1.11611	-0.07723
			C	-2.75097	-1.32682	-0.05242
			H	-1.36077	2.266742	0.081112
			H	-0.71647	-1.96459	-0.14809
			H	-3.13971	-2.33518	-0.09709
			C	0.570714	0.464986	-0.04011
			H	0.85336	1.511257	-0.10459
			C	1.56458	-0.42546	0.025359
			H	1.417965	-1.49213	0.112357

			C	2.953967	0.051045	-0.00274
			O	3.832665	-0.97052	0.120395
			H	4.722343	-0.59444	0.101634
			O	3.316898	1.196029	-0.11634
			H	-3.7996	1.889767	0.131596
			H	-4.69435	-0.416	0.044618
		SC2	Symbol	X	Y	Z
			C	-3.62605	-0.2475	0.024847
			C	-3.12522	1.046382	0.072243
			C	-1.75367	1.258755	0.044469
			C	-0.86481	0.183014	-0.02311
			C	-1.38213	-1.11615	-0.07511
			C	-2.75022	-1.32745	-0.05115
			H	-1.36174	2.26739	0.079208
			H	-0.71523	-1.9645	-0.14392
			H	-3.13846	-2.33609	-0.09472
			C	0.570444	0.466475	-0.03882
			H	0.851336	1.513344	-0.10075
			C	1.564451	-0.42435	0.024166
			H	1.41714	-1.49123	0.108056
			C	2.953991	0.050486	-0.00273
			O	3.83143	-0.97128	0.117616
			H	4.722898	-0.59849	0.099692
			O	3.318364	1.196249	-0.11358
			H	-3.80076	1.889193	0.128122
			H	-4.69421	-0.41736	0.043182
		SC3	Symbol	X	Y	Z
			C	-3.62486	-0.25275	0.020342
			C	-3.1287	1.043968	0.060812
			C	-1.75723	1.261258	0.037924
			C	-0.86504	0.186823	-0.01917
			C	-1.37682	-1.11554	-0.06321
			C	-2.74487	-1.33106	-0.04361
			H	-1.36815	2.271316	0.067659
			H	-0.70639	-1.96214	-0.12066
			H	-3.12969	-2.34145	-0.08054
			C	0.569402	0.47493	-0.03221
			H	0.843106	1.524167	-0.08345
			C	1.563012	-0.41842	0.020325
			H	1.410586	-1.48585	0.090021
			C	2.953834	0.047807	-0.00191
			O	3.82469	-0.97582	0.099397
			H	4.723888	-0.6178	0.083263
			O	3.324634	1.197201	-0.09632
			H	-3.80764	1.884817	0.1079
			H	-4.69269	-0.42627	0.035373
		SC4	Symbol	X	Y	Z
			C	-3.62509	0.252516	0.019003

			Symbol	X	Y	Z
			C	-3.12881	-1.04439	0.057969
			C	-1.7571	-1.26144	0.036594
			C	-0.86504	-0.18648	-0.01769
			C	-1.3768	1.116231	-0.0598
			C	-2.74509	1.331293	-0.0417
			H	-1.36768	-2.27143	0.065032
			H	-0.70679	1.963442	-0.11389
			H	-3.13009	2.341718	-0.07704
			C	0.569273	-0.47532	-0.02982
			H	0.840719	-1.52538	-0.07572
			C	1.563362	0.418216	0.017325
			H	1.41069	1.486054	0.080829
			C	2.954096	-0.04711	-0.00236
			O	3.824219	0.975793	0.096135
			H	4.725071	0.620783	0.082129
			O	3.325288	-1.1979	-0.09289
			H	-3.80772	-1.88546	0.102646
			H	-4.69301	0.425914	0.032908
	Ferulic Acid	Gas	Symbol	X	Y	Z
			C	-2.49163	-1.07705	0.000001
			C	-2.19024	0.2916	0.000023
			C	-0.87384	0.710551	0.000023
			C	0.169783	-0.2281	0.000001
			C	-0.14513	-1.58668	-0.00002
			C	-1.46584	-2.00782	-0.00002
			H	-0.63061	1.763389	0.000041
			H	0.641631	-2.32691	-3.5E-05
			H	-1.72418	-3.0571	-3.5E-05
			O	-3.78016	-1.48456	0.000001
			H	-4.34096	-0.70064	0.000017
			O	-3.29001	1.101217	0.000044
			C	-3.07277	2.504988	0.000057
			H	-2.52585	2.807208	-0.89398
			H	-4.05699	2.960975	0.000068
			H	-2.52583	2.807189	0.894093
			C	1.539738	0.2741	0.000001
			H	1.656275	1.353241	0.000034
			C	2.669149	-0.44198	-3.6E-05
			H	2.701067	-1.52123	-7.2E-05
			C	3.958902	0.262539	-2.7E-05
			O	4.997927	-0.60192	-6.6E-05
			H	5.80279	-0.07007	-5.8E-05
			O	4.123984	1.456644	0.00001
		Liquid	Symbol	X	Y	Z
			C	-2.48533	-1.07821	0
			C	-2.19474	0.292945	0.000022
			C	-0.87712	0.712492	0.000027
			C	0.166924	-0.22703	0.000008

			C	-0.13859	-1.58958	-1.3E-05
			C	-1.46014	-2.01037	-1.6E-05
			H	-0.63434	1.765457	0.000042
			H	0.647689	-2.33086	-2.4E-05
			H	-1.71261	-3.06185	-3.1E-05
			O	-3.78205	-1.50042	-5E-06
			H	-4.36112	-0.7261	0.000004
			O	-3.28984	1.10647	0.000038
			C	-3.05837	2.519767	0.000038
			H	-2.50991	2.81284	-0.8946
			H	-4.04073	2.979452	0.000085
			H	-2.50983	2.812829	0.894635
			C	1.532571	0.285263	0.000014
			H	1.624061	1.366529	0.000074
			C	2.663652	-0.43383	-4.8E-05
			H	2.686354	-1.5139	-0.00012
			C	3.954502	0.253455	-0.00002
			O	4.988807	-0.60838	-0.00012
			H	5.815796	-0.10339	-8.7E-05
			O	4.129882	1.458519	0.000079
		SC1	Symbol	X	Y	Z
			C	-2.18827	0.292088	0.000011
			C	-0.87195	0.710922	0.000013
			C	0.171004	-0.22874	0.000003
			C	-0.14263	-1.58805	-4E-06
			C	-1.46336	-2.0082	-6E-06
			H	-0.62887	1.764053	0.000019
			H	0.642938	-2.33025	-4E-06
			H	-1.7234	-3.05765	-9E-06
			O	-3.77949	-1.4901	0
			H	-4.35119	-0.71285	0.00001
			O	-3.28938	1.101642	0.000019
			C	-3.07731	2.507007	0.000059
			H	-2.53504	2.81444	-0.89545
			H	-4.06475	2.957734	0.0001
			H	-2.53499	2.814383	0.895556
			C	1.53985	0.27408	0.000004
			H	1.654248	1.353865	0.000059
			C	2.669632	-0.44248	-5.4E-05
			H	2.703192	-1.52195	-0.00012
			C	3.955275	0.262428	-2.4E-05
			O	4.999762	-0.59911	-0.00008
			H	5.809883	-0.07301	-4.6E-05
			O	4.118669	1.459257	0.000043
		SC2	Symbol	X	Y	Z
			C	-2.48676	-1.07619	0.000001
			C	-2.18876	0.292306	0.000013
			C	-0.87224	0.71101	0.000015

			Symbol	X	Y	Z
			C	0.170755	-0.22872	0.000003
			C	-0.14219	-1.58834	-7E-06
			C	-1.46303	-2.00837	-8E-06
			H	-0.6291	1.764129	0.000023
			H	0.643364	-2.33067	-9E-06
			H	-1.72281	-3.05798	-1.3E-05
			O	-3.77929	-1.4914	-1E-06
			H	-4.35311	-0.71539	0.000008
			O	-3.2891	1.102127	0.000024
			C	-3.07628	2.508395	0.000059
			H	-2.53384	2.814953	-0.89551
			H	-4.06373	2.959035	0.000101
			H	-2.53379	2.814902	0.895613
			C	1.53924	0.274939	0.000004
			H	1.651755	1.354885	0.000054
			C	2.669215	-0.44182	-5.1E-05
			H	2.701958	-1.52137	-0.00011
			C	3.955096	0.261664	-2.5E-05
			O	4.998736	-0.59951	-7.7E-05
			H	5.810952	-0.07597	-4.7E-05
			O	4.119399	1.459319	0.000037
		SC3	Symbol	X	Y	Z
			C	-2.48576	-1.07659	0.000001
			C	-2.1909	0.29319	0.000041
			C	-0.87363	0.711687	0.000041
			C	0.169659	-0.22833	0.000006
			C	-0.14021	-1.58931	-2.3E-05
			C	-1.46159	-2.00902	-2.6E-05
			H	-0.63034	1.764831	0.000067
			H	0.645249	-2.33207	-4.4E-05
			H	-1.71952	-3.05944	-5.2E-05
			O	-3.77854	-1.49695	-0.00001
			H	-4.36113	-0.72614	-1.3E-05
			O	-3.28823	1.10395	0.000081
			C	-3.07199	2.513981	0.000039
			H	-2.52842	2.816608	-0.89559
			H	-4.05934	2.964571	0.000074
			H	-2.52834	2.81665	0.895603
			C	1.536908	0.278805	0.000005
			H	1.641133	1.359491	0.000053
			C	2.667501	-0.4391	-0.00005
			H	2.696553	-1.51902	-0.0001
			C	3.954745	0.258246	-3.3E-05
			O	4.994411	-0.6013	-0.00009
			H	5.815349	-0.08858	-6.5E-05
			O	4.122404	1.459522	0.000025
		SC4	Symbol	X	Y	Z
			C	-2.48546	-1.07676	0.000001

			C	-2.19154	0.293374	0.000045
			C	-0.8741	0.711984	0.000045
			C	0.169386	-0.22802	0.000008
			C	-0.1396	-1.58941	-2.5E-05
			C	-1.46107	-2.00916	-2.9E-05
			H	-0.63096	1.765204	0.000073
			H	0.645856	-2.33223	-4.8E-05
			H	-1.71825	-3.05987	-5.7E-05
			O	-3.77826	-1.49856	-1.3E-05
			H	-4.36319	-0.72914	-2.1E-05
			O	-3.28816	1.104255	0.000088
			C	-3.07119	2.515311	0.000037
			H	-2.52733	2.817075	-0.89557
			H	-4.05858	2.965794	0.000077
			H	-2.52724	2.817127	0.895566
			C	1.536378	0.28002	0.000006
			H	1.638268	1.360924	0.000052
			C	2.66709	-0.43828	-4.8E-05
			H	2.695086	-1.51833	-0.0001
			C	3.954843	0.257286	-3.4E-05
			O	4.993237	-0.60183	-9.1E-05
			H	5.816519	-0.09207	-6.8E-05
			O	4.123355	1.459568	0.000022
	Methanol	Gas	Symbol	X	Y	Z
			C	0.665682	-0.0205	0
			H	1.081635	0.983201	0
			H	1.019684	-0.54433	0.890721
			H	1.019684	-0.54433	-0.89072
			O	-0.7461	0.121947	0
			H	-1.14628	-0.7471	0
		Liquid	Symbol	X	Y	Z
			C	-0.04804	0.67115	0
			H	-1.08649	0.991494	0
			H	0.446507	1.060122	0.890311
			H	0.446507	1.060122	-0.89031
			O	-0.04804	-0.76034	0
			H	0.866074	-1.05589	0
		SC1	Symbol	X	Y	Z
			C	-0.04666	0.664902	0
			H	-1.08713	0.980406	0
			H	0.441102	1.067036	0.891235
			H	0.441102	1.067036	-0.89124
			O	-0.04666	-0.75441	0
			H	0.858198	-1.06861	0
		SC2	Symbol	X	Y	Z
			C	-0.04678	0.66566	0
			H	-1.0871	0.981656	0
			H	0.441625	1.066491	0.89128

			Symbol	X	Y	Z
			H	0.441625	1.066491	-0.89128
			O	-0.04678	-0.75502	0
			H	0.85873	-1.06843	0
		SC3	Symbol	X	Y	Z
			C	-0.04726	0.668139	0
			H	-1.08712	0.985767	0
			H	0.443921	1.06366	0.891181
			H	0.443921	1.06366	-0.89118
			O	-0.04726	-0.75687	0
			H	0.860914	-1.06692	0
		SC4	Symbol	X	Y	Z
			C	-0.0474	0.668829	0
			H	-1.08715	0.98698	0
			H	0.444592	1.062919	0.891126
			H	0.444592	1.062919	-0.89113
			O	-0.0474	-0.75743	0
			H	0.861598	-1.06632	0
6	Furan	Gas	Symbol	X	Y	Z
			C	0	-0.34658	1.09038
			C	0	0.954167	0.717376
			C	0	0.954167	-0.71738
			C	0	-0.34658	-1.09038
			O	0	-1.15265	0
			H	0.000004	-0.84111	2.044154
			H	-1E-06	1.806172	1.373898
			H	-1E-06	1.806172	-1.3739
			H	0.000004	-0.84111	-2.04415
		Liquid	Symbol	X	Y	Z
			C	0.000001	-0.34342	1.098712
			C	0.000001	0.953811	0.718935
			C	0.000001	0.953811	-0.71894
			C	0.000001	-0.34342	-1.09871
			O	-7E-06	-1.15805	0
			H	0.000009	-0.8386	2.053425
			H	0.000004	1.808486	1.37384
			H	0.000004	1.808486	-1.37384
			H	0.000009	-0.8386	-2.05343
		SC1	Symbol	X	Y	Z
			C	0	-0.34472	1.09359
			C	0	0.953924	0.717693
			C	0	0.953924	-0.71769
			C	0	-0.34472	-1.09359
			O	0	-1.15561	0
			H	0.000005	-0.83993	2.047842
			H	0	1.807159	1.373751
			H	0	1.807159	-1.37375
			H	0.000005	-0.83993	-2.04784
		SC2	Symbol	X	Y	Z

			Symbol	X	Y	Z
			C	0	-0.34464	1.094071
			C	0	0.953903	0.717821
			C	0	0.953903	-0.71782
			C	0	-0.34464	-1.09407
			O	-1E-06	-1.15572	0
			H	0.000005	-0.83998	2.048368
			H	0.000001	1.807295	1.373859
			H	0.000001	1.807295	-1.37386
			H	0.000005	-0.83998	-2.04837
		SC3	Symbol	X	Y	Z
			C	0	-0.34432	1.096163
			C	0	0.953814	0.718378
			C	0	0.953814	-0.71838
			C	0	-0.34432	-1.09616
			O	-3E-06	-1.15621	0
			H	0.000006	-0.84005	2.050736
			H	0.000003	1.807912	1.374251
			H	0.000003	1.807912	-1.37425
			H	0.000006	-0.84005	-2.05074
		SC4	Symbol	X	Y	Z
			C	0	-0.34424	1.096705
			C	0	0.953785	0.718528
			C	0	0.953785	-0.71853
			C	0	-0.34424	-1.09671
			O	-3E-06	-1.15633	0
			H	0.000007	-0.84002	2.051393
			H	0.000003	1.808064	1.374363
			H	0.000003	1.808064	-1.37436
			H	0.000007	-0.84002	-2.05139
	Furfural	Gas	Symbol	X	Y	Z
			C	-1.97472	0.414306	-5.9E-05
			C	-0.76882	1.171636	-4.9E-05
			C	0.250556	0.264663	-3E-06
			O	-0.24778	-0.99656	0.000015
			H	-2.13648	-1.81433	-0.00001
			H	-2.98548	0.781375	-9.6E-05
			H	-0.66193	2.243039	-7.3E-05
			C	1.694104	0.466242	0.000033
			H	1.985853	1.529795	0.000017
			O	2.512792	-0.41644	0.000078
		Liquid	Symbol	X	Y	Z
			C	-1.58614	-0.89338	-0.00002
			C	-1.97076	0.411212	-6.3E-05
			C	-0.77078	1.173786	-4.8E-05
			C	0.256532	0.275266	0.000003
			O	-0.23773	-1.00234	0.000021
			H	-2.13627	-1.81843	-1.3E-05
			H	-2.98326	0.77565	-0.0001

			H	-0.66622	2.246128	-7.1E-05
			C	1.682255	0.479494	0.000031
			H	1.988049	1.532083	0.000012
			O	2.50411	-0.42437	0.000074
		SC1	Symbol	X	Y	Z
			C	-1.5834	-0.89326	-1.7E-05
			C	-1.9742	0.408733	-6.1E-05
			C	-0.77325	1.172754	-4.8E-05
			C	0.252418	0.273546	0.000002
			O	-0.23968	-0.99555	0.000022
			H	-2.12821	-1.82109	-7E-06
			H	-2.98715	0.771497	-0.0001
			H	-0.67461	2.245521	-7.6E-05
			C	1.691399	0.472273	0.000034
			H	1.995656	1.530914	0.000027
			O	2.504248	-0.42084	0.000065
		SC2	Symbol	X	Y	Z
			C	-1.58322	-0.89354	-1.7E-05
			C	-1.97394	0.408622	-0.00006
			C	-0.77328	1.172903	-4.7E-05
			C	0.252756	0.273927	0.000001
			O	-0.23921	-0.99578	0.000022
			H	-2.12807	-1.82145	-7E-06
			H	-2.98702	0.771251	-0.0001
			H	-0.6747	2.245752	-7.6E-05
			C	1.690502	0.472998	0.000034
			H	1.995436	1.530912	0.000027
			O	2.503895	-0.42121	0.000066
		SC3	Symbol	X	Y	Z
			C	-1.58171	-0.8952	-1.7E-05
			C	-1.97276	0.40746	-5.7E-05
			C	-0.77394	1.173647	-4.7E-05
			C	0.254358	0.276512	-3E-06
			O	-0.23625	-0.99657	0.000018
			H	-2.12657	-1.82354	-5E-06
			H	-2.98661	0.768909	-0.0001
			H	-0.67596	2.246851	-7.8E-05
			C	1.686441	0.476849	0.000035
			H	1.994842	1.531399	0.00003
			O	2.501252	-0.42334	0.000068
		SC4	Symbol	X	Y	Z
			C	-1.58137	-0.89579	-1.8E-05
			C	-1.97262	0.406974	-6.5E-05
			C	-0.7745	1.17394	-4.8E-05
			C	0.254698	0.277624	0.000007
			O	-0.23538	-0.99683	0.000025
			H	-2.12627	-1.82426	-0.00001
			H	-2.98675	0.767949	-0.00011

			H	-0.6768	2.24727	-7.3E-05
			C	1.685824	0.478163	0.000033
			H	1.995562	1.531866	0.00002
			O	2.500637	-0.42421	0.000065
7	Butanol	Gas	Symbol	X	Y	Z
			C	2.516158	0.075147	0
			H	2.615096	0.710442	0.881283
			H	2.615096	0.710442	-0.88128
			H	3.343947	-0.63353	0
			C	1.167754	-0.63981	0
			H	1.097486	-1.28896	-0.87668
			H	1.097486	-1.28896	0.876682
			C	0	0.342408	0
			H	0.053425	0.989239	-0.87856
			H	0.053425	0.989239	0.878556
			C	-1.34092	-0.36018	0
			H	-1.42632	-0.99586	0.887245
			H	-1.42632	-0.99586	-0.88725
			O	-2.35822	0.636015	0
			H	-3.21555	0.210297	0
		Liquid	Symbol	X	Y	Z
			C	2.516314	0.106049	0
			H	2.610335	0.741256	0.882585
			H	2.610335	0.741256	-0.88259
			H	3.349986	-0.59673	0
			C	1.176483	-0.6222	0
			H	1.109994	-1.26969	-0.87773
			H	1.109994	-1.26969	0.877725
			C	0	0.349484	0
			H	0.05354	0.993333	-0.88186
			H	0.05354	0.993333	0.881855
			C	-1.32682	-0.37595	0
			H	-1.41247	-1.00761	0.88695
			H	-1.41247	-1.00761	-0.88695
			O	-2.38074	0.60018	0
			H	-3.22273	0.136397	0
		SC1	Symbol	X	Y	Z
			C	2.515251	0.068002	0
			H	2.619835	0.702676	0.881797
			H	2.619835	0.702676	-0.8818
			H	3.341132	-0.64411	0
			C	1.166792	-0.64443	0
			H	1.097775	-1.29387	-0.87694
			H	1.097775	-1.29387	0.876941
			C	0	0.337898	0
			H	0.053531	0.985087	-0.87899
			H	0.053531	0.985087	0.878993
			C	-1.34344	-0.35801	0

			H	-1.43939	-0.99132	0.887787
			H	-1.43939	-0.99132	-0.88779
			O	-2.35245	0.64806	0
			H	-3.21661	0.233703	0
		SC2	Symbol	X	Y	Z
			C	2.499848	0.294765	-0.00104
			H	2.547324	0.943325	0.875681
			H	2.550795	0.929117	-0.88789
			H	3.385272	-0.34207	0.005777
			C	1.2185	-0.5314	0.003145
			H	1.205588	-1.18911	-0.87035
			H	1.205342	-1.17975	0.883562
			C	-0.0303	0.345192	-0.00109
			H	-0.03253	0.993156	-0.88114
			H	-0.03685	0.99477	0.877752
			C	-1.30028	-0.47627	-0.003
			H	-1.33114	-1.12075	0.881324
			H	-1.33389	-1.1126	-0.89307
			O	-2.40784	0.421481	0.002885
			H	-3.22377	-0.08171	-0.00284
		SC3	Symbol	X	Y	Z
			C	2.501048	0.29304	0.00627
			H	2.560753	0.891625	0.917335
			H	2.539887	0.975892	-0.84477
			H	3.38545	-0.34426	-0.03434
			C	1.218422	-0.53119	-0.01841
			H	1.202269	-1.15564	-0.91571
			H	1.203615	-1.21078	0.838177
			C	-0.02871	0.348038	0.00649
			H	-0.04156	1.000961	-0.87022
			H	-0.01714	0.99082	0.890653
			C	-1.29772	-0.47464	0.017095
			H	-1.34093	-1.08948	0.920656
			H	-1.32399	-1.13729	-0.85292
			O	-2.41272	0.419666	-0.01772
			H	-3.22485	-0.09072	0.024224
		SC4	Symbol	X	Y	Z
			C	2.501334	0.292721	0.006189
			H	2.56096	0.892222	0.916741
			H	2.540598	0.974525	-0.84576
			H	3.385481	-0.34511	-0.03347
			C	1.218457	-0.53117	-0.01787
			H	1.201818	-1.1563	-0.91466
			H	1.202885	-1.20969	0.839513
			C	-0.02833	0.34869	0.006084
			H	-0.03972	1.001014	-0.87124
			H	-0.01617	0.991505	0.890356
			C	-1.29702	-0.47441	0.016544

			Symbol	X	Y	Z
			H	-1.34069	-1.08953	0.919602
			H	-1.32443	-1.136	-0.85396
			O	-2.414	0.419432	-0.0171
			H	-3.22531	-0.09312	0.023979
	tetrahydrofuran	Gas	Symbol	X	Y	Z
			C	0.731348	1.044844	-0.00081
			C	-0.8099	0.974073	-0.10293
			C	-1.10344	-0.49737	0.198086
			C	1.150866	-0.43446	0.121367
			H	1.16041	1.505318	-0.88785
			H	1.05837	1.617065	0.865348
			H	-1.13613	1.215592	-1.11278
			H	-1.3122	1.646659	0.589561
			H	-1.19567	-0.6623	1.278365
			H	-1.9876	-0.88679	-0.29932
			H	1.389829	-0.68185	1.16103
			H	1.992714	-0.70661	-0.50934
			O	0.027123	-1.1962	-0.29742
		Liquid	Symbol	X	Y	Z
			C	0.773664	1.009468	-0.05341
			C	-0.77402	1.009175	-0.05369
			C	-1.13333	-0.46306	0.166162
			C	1.133528	-0.46275	0.165952
			H	1.157298	1.356941	-1.01036
			H	1.191458	1.636657	0.730988
			H	-1.1574	1.356007	-1.01098
			H	-1.19239	1.63658	0.730223
			H	-1.27313	-0.67657	1.229437
			H	-2.00791	-0.79493	-0.38659
			H	1.27378	-0.67648	1.229118
			H	2.008026	-0.79416	-0.3872
			O	0.00015	-1.20013	-0.30934
		SC1	Symbol	X	Y	Z
			C	0.773546	1.010715	-0.05083
			C	-0.77406	1.010227	-0.05166
			C	-1.12746	-0.46767	0.162696
			C	1.127788	-0.46729	0.162064
			H	1.157763	1.361099	-1.00686
			H	1.192605	1.640036	0.732259
			H	-1.1574	1.358863	-1.00868
			H	-1.19448	1.640421	0.73
			H	-1.28305	-0.68179	1.226257
			H	-1.99833	-0.80168	-0.39568
			H	1.284525	-0.68209	1.225307
			H	1.998291	-0.80049	-0.39736
			O	0.000144	-1.19379	-0.30486
		SC2	Symbol	X	Y	Z
			C	0.773571	1.010635	-0.05091

			Symbol	X	Y	Z
			C	-0.77409	1.01014	-0.05175
			C	-1.12803	-0.46734	0.162802
			C	1.128361	-0.46696	0.162158
			H	1.157754	1.361023	-1.00697
			H	1.192536	1.639672	0.732447
			H	-1.15738	1.35875	-1.00883
			H	-1.19444	1.640065	0.730148
			H	-1.28296	-0.68171	1.226238
			H	-1.9989	-0.80123	-0.39564
			H	1.284465	-0.68202	1.225272
			H	1.998864	-0.80002	-0.39735
			O	0.000145	-1.19417	-0.30489
		SC3	Symbol	X	Y	Z
			C	0.773954	1.009992	-0.05183
			C	-0.77397	1.00996	-0.052
			C	-1.13126	-0.4654	0.163427
			C	1.131283	-0.46542	0.163302
			H	1.15762	1.359425	-1.00859
			H	1.192992	1.638445	0.731885
			H	-1.15744	1.359084	-1.00894
			H	-1.19321	1.638621	0.731443
			H	-1.28262	-0.68097	1.226354
			H	-2.00235	-0.79831	-0.39529
			H	1.282846	-0.68114	1.226169
			H	2.002279	-0.79826	-0.39561
			O	-2.4E-05	-1.19646	-0.3056
		SC4	Symbol	X	Y	Z
			C	0.774014	1.009816	-0.05205
			C	-0.77397	1.009831	-0.05214
			C	-1.13209	-0.46491	0.163672
			C	1.132082	-0.46496	0.163597
			H	1.157685	1.35916	-1.00887
			H	1.192923	1.637994	0.731914
			H	-1.15752	1.359009	-1.00908
			H	-1.19298	1.638151	0.731654
			H	-1.2823	-0.68067	1.226484
			H	-2.00334	-0.79761	-0.39495
			H	1.282396	-0.68082	1.226375
			H	2.003262	-0.79767	-0.39513
			O	-4.2E-05	-1.19703	-0.30586
8	Xylose	Gas	Symbol	X	Y	Z
			O	0.750504	-1.6357	-0.23355
			O	-1.46771	1.839158	-0.32401
			O	1.297218	1.92195	0.326699
			O	-2.71719	-0.65012	0.202906
			O	2.735113	-0.52689	-0.1141
			C	-0.7446	0.742043	0.204944
			C	0.705981	0.759552	-0.22083

			C	-1.40235	-0.52672	-0.2965
			C	1.39808	-0.4992	0.286696
			C	-0.60661	-1.72749	0.192622
			H	-0.79142	0.75437	1.300907
			H	0.763755	0.769351	-1.31605
			H	-1.40236	-0.50999	-1.39146
			H	1.394228	-0.51189	1.381268
			H	-0.65916	-1.77674	1.285034
			H	-1.00685	-2.64732	-0.22189
			H	-1.01249	2.645794	-0.06709
			H	2.242875	1.884165	0.156955
			H	-3.17031	0.178577	0.022967
			H	2.755172	-0.86272	-1.01588
		Liquid	Symbol	X	Y	Z
			O	0.714974	-1.65319	-0.25815
			O	-1.42395	1.866318	-0.358
			O	1.33296	1.891524	0.384628
			O	-2.73451	-0.61956	0.217477
			O	2.736409	-0.59786	-0.12251
			C	-0.73605	0.753929	0.203011
			C	0.725969	0.74896	-0.20189
			C	-1.41089	-0.50973	-0.29098
			C	1.39784	-0.53106	0.280714
			C	-0.63568	-1.7193	0.202453
			H	-0.8025	0.787063	1.295366
			H	0.803746	0.790056	-1.29303
			H	-1.42408	-0.50455	-1.38402
			H	1.390919	-0.5819	1.371546
			H	-0.65537	-1.75314	1.295812
			H	-1.06292	-2.63507	-0.19494
			H	-1.05062	2.671002	0.016859
			H	2.209339	2.004574	0.002223
			H	-3.23805	0.13853	-0.0979
			H	2.775344	-0.47126	-1.07921
		SC1	Symbol	X	Y	Z
			O	0.742605	-1.63742	-0.23479
			O	-1.45625	1.841721	-0.34538
			O	1.300154	1.915868	0.347987
			O	-2.71768	-0.64573	0.213117
			O	2.73715	-0.54091	-0.1237
			C	-0.74322	0.746482	0.202578
			C	0.710975	0.758754	-0.21443
			C	-1.40372	-0.52255	-0.29444
			C	1.399201	-0.50383	0.286421
			C	-0.61367	-1.7256	0.194088
			H	-0.80078	0.77147	1.297549
			H	0.774932	0.781029	-1.30893
			H	-1.41192	-0.50991	-1.38929

			H	1.407509	-0.52326	1.380392
			H	-0.6664	-1.77927	1.286518
			H	-1.01735	-2.64379	-0.22255
			H	-1.03433	2.656091	-0.05507
			H	2.243173	1.906427	0.157289
			H	-3.19408	0.162449	-0.00024
			H	2.753973	-0.80904	-1.04889
		SC2	Symbol	X	Y	Z
			O	0.740956	-1.63792	-0.23691
			O	-1.4546	1.842687	-0.3487
			O	1.301746	1.914422	0.351846
			O	-2.7184	-0.64476	0.214894
			O	2.737494	-0.54463	-0.12359
			C	-0.74298	0.747241	0.201927
			C	0.712091	0.758654	-0.21378
			C	-1.40419	-0.52207	-0.29381
			C	1.39944	-0.50526	0.285561
			C	-0.61468	-1.72525	0.195
			H	-0.80171	0.774094	1.296651
			H	0.777105	0.783307	-1.30807
			H	-1.41369	-0.51059	-1.38854
			H	1.407211	-0.52671	1.379316
			H	-0.66452	-1.77753	1.287573
			H	-1.01986	-2.64347	-0.22017
			H	-1.04072	2.658008	-0.04907
			H	2.24365	1.910447	0.154747
			H	-3.1984	0.159578	-0.00559
			H	2.755126	-0.78544	-1.05653
		SC3	Symbol	X	Y	Z
			O	0.733157	-1.64037	-0.2467
			O	-1.44563	1.848454	-0.36031
			O	1.309168	1.908059	0.366305
			O	-2.7215	-0.63976	0.222905
			O	2.738725	-0.56406	-0.12057
			C	-0.74111	0.750215	0.199354
			C	0.717337	0.757659	-0.21177
			C	-1.40652	-0.51925	-0.29194
			C	1.400094	-0.51203	0.282257
			C	-0.61993	-1.72386	0.196961
			H	-0.80402	0.78357	1.293016
			H	0.787356	0.79125	-1.30495
			H	-1.42255	-0.51174	-1.38611
			H	1.402275	-0.54296	1.375096
			H	-0.65798	-1.77093	1.290026
			H	-1.03158	-2.64164	-0.21318
			H	-1.07299	2.664928	-0.01155
			H	2.242274	1.935073	0.129881
			H	-3.2194	0.143206	-0.03489

			H	2.765954	-0.66578	-1.07956
		SC4	Symbol	X	Y	Z
			O	0.730377	-1.64156	-0.24923
			O	-1.44188	1.851096	-0.36269
			O	1.312365	1.905526	0.370195
			O	-2.72265	-0.63745	0.225078
			O	2.738668	-0.57049	-0.11967
			C	-0.74023	0.751292	0.199013
			C	0.719101	0.756843	-0.21093
			C	-1.40743	-0.51769	-0.2916
			C	1.400029	-0.51451	0.281641
			C	-0.62229	-1.72333	0.196889
			H	-0.80432	0.786138	1.29237
			H	0.790682	0.792571	-1.30374
			H	-1.42539	-0.5108	-1.38558
			H	1.400289	-0.54844	1.374226
			H	-0.65764	-1.7696	1.290025
			H	-1.03602	-2.64052	-0.21265
			H	-1.08111	2.666859	0.000434
			H	2.240118	1.945842	0.114662
			H	-3.227	0.136684	-0.04709
			H	2.770231	-0.63133	-1.08228
9	cyclohexanone	Gas	Symbol	X	Y	Z
			C	-0.4291	1.062667	0
			C	0.170574	0.514458	1.275747
			C	0.170574	-1.02194	1.25678
			C	0.856997	-1.55477	0
			C	0.170574	-1.02194	-1.25678
			C	0.170574	0.514458	-1.27575
			H	0.662627	-1.39741	2.154182
			H	1.204874	0.869011	1.33506
			H	-0.38397	0.9178	2.120318
			H	1.906711	-1.24451	0
			H	0.844324	-2.64534	0
			H	0.662627	-1.39741	-2.15418
			H	-0.86206	-1.37981	-1.28304
			H	1.204874	0.869011	-1.33506
			H	-0.38397	0.9178	-2.12032
			H	-0.86206	-1.37981	1.283038
			O	-1.33189	1.864122	0
		Liquid	Symbol	X	Y	Z
			C	-0.41931	1.054738	0
			C	0.165491	0.511593	1.271866
			C	0.165491	-1.02602	1.254692
			C	0.853986	-1.55673	0
			C	0.165491	-1.02602	-1.25469
			C	0.165491	0.511593	-1.27187
			H	0.661935	-1.38983	2.153783

			H	1.200728	0.864056	1.320377
			H	-0.38326	0.913557	2.121258
			H	1.901354	-1.24108	0
			H	0.839836	-2.64718	0
			H	0.661935	-1.38983	-2.15378
			H	-0.86641	-1.38472	-1.28194
			H	1.200728	0.864056	-1.32038
			H	-0.38326	0.913557	-2.12126
			H	-0.86641	-1.38472	1.281942
			O	-1.31838	1.883399	0
		SC1	Symbol	X	Y	Z
			C	-0.41773	1.064778	0
			C	0.161597	0.506195	1.277046
			C	0.161597	-1.02937	1.256391
			C	0.846395	-1.5631	0
			C	0.161597	-1.02937	-1.25639
			C	0.161597	0.506195	-1.27705
			H	0.65511	-1.40372	2.153911
			H	1.194597	0.863022	1.349705
			H	-0.40085	0.909083	2.117466
			H	1.898237	-1.25889	0
			H	0.829837	-2.65414	0
			H	0.65511	-1.40372	-2.15391
			H	-0.87081	-1.38803	-1.28588
			H	1.194597	0.863022	-1.34971
			H	-0.40085	0.909083	-2.11747
			H	-0.87081	-1.38803	1.285882
			O	-1.29182	1.90254	0
		SC2	Symbol	X	Y	Z
			C	-0.4171	1.064277	0
			C	0.161284	0.506078	1.276813
			C	0.161284	-1.02962	1.256273
			C	0.846198	-1.56336	0
			C	0.161284	-1.02962	-1.25627
			C	0.161284	0.506078	-1.27681
			H	0.655054	-1.40315	2.153967
			H	1.194387	0.862807	1.348503
			H	-0.40066	0.908862	2.117655
			H	1.897983	-1.25905	0
			H	0.829173	-2.65439	0
			H	0.655054	-1.40315	-2.15397
			H	-0.87111	-1.38826	-1.28576
			H	1.194387	0.862807	-1.3485
			H	-0.40066	0.908862	-2.11766
			H	-0.87111	-1.38826	1.285756
			O	-1.29099	1.903729	0
		SC3	Symbol	X	Y	Z
			C	-0.41434	1.061894	0

			Symbol	X	Y	Z
			C	0.159942	0.505604	1.275719
			C	0.159942	-1.03069	1.255678
			C	0.845831	-1.56387	0
			C	0.159942	-1.03069	-1.25568
			C	0.159942	0.505604	-1.27572
			H	0.654819	-1.40062	2.15413
			H	1.193531	0.861688	1.343291
			H	-0.39997	0.907892	2.118374
			H	1.896964	-1.25773	0
			H	0.828163	-2.65494	0
			H	0.654819	-1.40062	-2.15413
			H	-0.87235	-1.38941	-1.28485
			H	1.193531	0.861688	-1.34329
			H	-0.39997	0.907892	-2.11837
			H	-0.87235	-1.38941	1.284847
			O	-1.28809	1.908313	0
		SC4	Symbol	X	Y	Z
			C	-0.41382	1.060981	0
			C	0.159565	0.505493	1.275081
			C	0.159565	-1.03098	1.255416
			C	0.84588	-1.56398	0
			C	0.159565	-1.03098	-1.25542
			C	0.159565	0.505493	-1.27508
			H	0.654705	-1.39976	2.154133
			H	1.193367	0.861305	1.340962
			H	-0.3993	0.907739	2.118516
			H	1.896739	-1.25706	0
			H	0.828255	-2.65506	0
			H	0.654705	-1.39976	-2.15413
			H	-0.87268	-1.38981	-1.28435
			H	1.193367	0.861305	-1.34096
			H	-0.3993	0.907739	-2.11852
			H	-0.87268	-1.38981	1.284352
			O	-1.28739	1.909634	0
	Guaiacol	Gas	Symbol	X	Y	Z
			C	-2.28418	-0.5993	0.000122
			C	-1.83478	0.719197	0.000108
			C	-0.47893	0.984476	-3.9E-05
			C	0.439786	-0.07372	-0.00017
			C	-0.00531	-1.38491	-0.00018
			C	-1.3765	-1.64513	-2.8E-05
			H	-3.34598	-0.80081	0.000247
			H	-2.52209	1.55334	0.000232
			H	0.696968	-2.20505	-0.00034
			H	-1.72169	-2.66906	-4.6E-05
			O	-0.03228	2.266575	-6E-06
			H	0.930571	2.243042	-0.00017
			O	1.748403	0.327539	-0.00018

			Symbol	X	Y	Z
			C	2.742846	-0.68363	0.000253
			H	3.697511	-0.16794	0.001428
			H	2.658209	-1.30462	0.89336
			H	2.659958	-1.30376	-0.8936
		Liquid	Symbol	X	Y	Z
			C	-2.28184	-0.62138	-0.01152
			C	-1.84464	0.702879	-0.01236
			C	-0.49064	0.978547	0.002136
			C	0.444294	-0.06548	0.016966
			C	0.009934	-1.38232	0.018192
			C	-1.3591	-1.65619	0.003623
			H	-3.34179	-0.83358	-0.02385
			H	-2.54486	1.527317	-0.02593
			H	0.722651	-2.19374	0.031013
			H	-1.69178	-2.68473	0.004394
			O	-0.05998	2.281937	-0.00011
			H	0.906179	2.286675	-0.00029
			O	1.749816	0.339715	0.030393
			C	2.753807	-0.67812	-0.03154
			H	3.703561	-0.15422	-0.05216
			H	2.701471	-1.31731	0.849184
			H	2.634968	-1.27128	-0.93766
		SC1	Symbol	X	Y	Z
			C	-2.28712	-0.5941	-0.01987
			C	-1.83361	0.722894	-0.02179
			C	-0.47729	0.981687	0.00344
			C	0.438158	-0.07747	0.030333
			C	-0.01069	-1.38721	0.031001
			C	-1.38227	-1.64231	0.005976
			H	-3.34981	-0.79228	-0.04037
			H	-2.51904	1.559012	-0.04529
			H	0.689684	-2.20918	0.052775
			H	-1.7313	-2.66549	0.007482
			O	-0.02527	2.269613	-0.00109
			H	0.938903	2.250615	0.004322
			O	1.748652	0.319807	0.055391
			C	2.7449	-0.68499	-0.0559
			H	3.695135	-0.16053	-0.09165
			H	2.727708	-1.34699	0.811572
			H	2.609196	-1.26158	-0.97234
		SC2	Symbol	X	Y	Z
			C	-2.28677	-0.59616	-0.0201
			C	-1.83439	0.72139	-0.02197
			C	-0.47823	0.98141	0.003515
			C	0.438715	-0.0766	0.030748
			C	-0.00918	-1.38687	0.031373
			C	-1.38058	-1.6434	0.005997
			H	-3.34931	-0.79534	-0.04088

			H	-2.52096	1.556683	-0.0457
			H	0.692216	-2.20799	0.053355
			H	-1.72844	-2.66704	0.00739
			O	-0.02804	2.270605	-0.00113
			H	0.936437	2.254712	0.003308
			O	1.748655	0.320913	0.056121
			C	2.74574	-0.68434	-0.05657
			H	3.69553	-0.1592	-0.09309
			H	2.729048	-1.34628	0.810812
			H	2.608641	-1.26021	-0.97314
		SC3	Symbol	X	Y	Z
			C	-2.28548	-0.60475	-0.02082
			C	-1.83777	0.715138	-0.02262
			C	-0.48215	0.980183	0.0037
			C	0.441046	-0.07309	0.032177
			C	-0.00293	-1.38564	0.032478
			C	-1.37363	-1.64802	0.006028
			H	-3.34745	-0.80812	-0.04237
			H	-2.52942	1.546767	-0.04719
			H	0.702607	-2.20336	0.055451
			H	-1.71655	-2.67362	0.007193
			O	-0.03979	2.274775	-0.00128
			H	0.925899	2.271984	-0.0019
			O	1.748661	0.325542	0.059402
			C	2.749888	-0.68135	-0.05916
			H	3.697879	-0.15316	-0.09398
			H	2.732183	-1.34621	0.805261
			H	2.609934	-1.25163	-0.97818
		SC4	Symbol	X	Y	Z
			C	-2.28511	-0.60714	-0.02089
			C	-1.8387	0.713404	-0.0227
			C	-0.48321	0.979863	0.003719
			C	0.441699	-0.07213	0.032465
			C	-0.00117	-1.38532	0.032592
			C	-1.37169	-1.64929	0.006001
			H	-3.34692	-0.81168	-0.04251
			H	-2.53186	1.54393	-0.04743
			H	0.705465	-2.20216	0.055488
			H	-1.71319	-2.67545	0.00707
			O	-0.04312	2.275844	-0.00129
			H	0.922866	2.276784	-0.00412
			O	1.748645	0.326879	0.060199
			C	2.751048	-0.68044	-0.05963
			H	3.698531	-0.15137	-0.09428
			H	2.733247	-1.34585	0.804194
			H	2.61042	-1.24962	-0.97906
	Hydrogen	Gas	Symbol	X	Y	Z
			H	0	0	0.369708

			H	0	0	-0.36971
		Liquid	Symbol	X	Y	Z
			H	0	0	0.370001
			H	0	0	-0.37
		SC1	Symbol	X	Y	Z
			H	0	0	0.369855
			H	0	0	-0.36986
		SC2	Symbol	X	Y	Z
			H	0	0	0.369902
			H	0	0	-0.3699
		SC3	Symbol	X	Y	Z
			H	0	0	0.370095
			H	0	0	-0.3701
		SC4	Symbol	X	Y	Z
			H	0	0	0.370145
			H	0	0	-0.37015
